# Quantitative assessment of single-cell whole genome amplification methods for detecting copy number variation using hippocampal neurons

**DOI:** 10.1038/srep11415

**Published:** 2015-06-19

**Authors:** Luwen Ning, Zhoufang Li, Guan Wang, Wen Hu, Qingming Hou, Yin Tong, Meng Zhang, Yao Chen, Li Qin, Xiaoping Chen, Heng-Ye Man, Pinghua Liu, Jiankui He

**Affiliations:** 1 Department of Biology, South University of Science and Technology of China, Shenzhen 518055, China; 2 Department of Biology, Boston University, Boston, MA 02215, USA; 3 Department of Chemistry, Boston University, Boston, MA 02215, USA; 4 State Key Laboratory of Respiratory Disease, Guangzhou Institute of Biomedicine and Health, Chinese Academy of Sciences, Guangzhou 510530, China

## Abstract

Single-cell genomic analysis has grown rapidly in recent years and finds widespread applications in various fields of biology, including cancer biology, development, immunology, pre-implantation genetic diagnosis, and neurobiology. To date, the amplification bias, amplification uniformity and reproducibility of the three major single cell whole genome amplification methods (GenomePlex WGA4, MDA and MALBAC) have not been systematically investigated using mammalian cells. In this study, we amplified genomic DNA from individual hippocampal neurons using three single-cell DNA amplification methods, and sequenced them at shallow depth. We then systematically evaluated the GC-bias, reproducibility, and copy number variations among individual neurons. Our results showed that single-cell genome sequencing results obtained from the MALBAC and WGA4 methods are highly reproducible and have a high success rate. The MALBAC displays significant biases towards high GC content. We then attempted to correct the GC bias issue by developing a bioinformatics pipeline, which allows us to call CNVs in single cell sequencing data, and chromosome level and sub-chromosomal level CNVs among individual neurons can be detected. We also proposed a metric to determine the CNV detection limits. Overall, MALBAC and WGA4 have better performance than MDA in detecting CNVs.

Interest in single-cell whole genome analysis is growing rapidly, especially for profiling rare or heterogeneous populations of cells. Single-cell whole genome sequencing has been applied to study cancer biology, cell development, neurobiology, and pre-implantation genetic diagnosis^1^,^2^,^3^,^4^. Single-nucleotide polymorphisms (SNPs) and copy number variations (CNVs) are two major types of genetic polymorphism contributing to the heterogeneity of cell populations. To detect SNPs in single cells, deep sequencing at >30X coverage is usually performed. For example, Hou *et al.*^5^ performed single cell exome sequencing on myeloproliferative neoplasm at 30X coverage and identified essential thrombocythemia-related candidate mutations. To detect CNVs, chromosome rearrangement, large-scale insertion/deletion, shallow sequencing to <1X coverage is usually performed. Shallow sequencing for identifying CNVs is a common practice reported by several researchers. For example, by sequencing 100 single neurons at about 0.04X coverage, McConnell *et al.*^2^ identified aneuploid neurons, as well as numerous subchromosomal CNVs in euploid neurons. While whole genome deep sequencing is still expensive, shallow sequencing for CNV detection can be used to study hundreds of cells with a reasonable budget. CNVs have important roles in human health and have been reported to be associated with various human diseases, such as tumors, autism, autoimmunity, systematic lupus, and erythematous. In this study, we will focus on three whole genome amplification methods for the detection of CNVs in single neurons.

The key to the success of identifying CNVs and large-scale rearrangement in individual neurons is amplification of the genetic materials from a single cell by a high-fidelity and low-bias method. Over the years, several single-cell whole genome amplification methods were reported. The first method is multiple displacement amplification (MDA). MDA is a non-PCR-based DNA amplification technique, which uses a high fidelity enzyme, typically Φ29 DNA polymerase, to amplify the target genome. Previous studies in other groups have reported mixed results of calling chromosome-level CNVs in single cell samples by MDA methods performed in eppendorf tubes. Gole *et al.* suggested that the MDA method performed in an eppendorf tube is not able to identify trisomy 21^6^. Cai *et al.* suggested that the MDA method was able to identify the sex chromosome in a male sample^7^. However, several studies have demonstrated that in microfluidic devices and nanoliter devices, the MDA method’s performance can be significantly improved compared to studies conducted in eppendorf tubes, and therefore is able to call chromosome level CNVs^1^,^8^. GenomePlex whole genome amplification (WGA4) is another single-cell whole genome amplification method, which is based on the PCR amplification of randomly fragmented genomic DNAs using universal oligonucleotides as primers. Recently, WGA4 was applied to analyze cancer cell CNVs^9^. The WGA4 method has also been used to study genomic diversity in neurons^2^. In 2012, Zong *et al.* described a third single-cell genome amplification method, the multiple annealing and looping-based amplification cycles (MALBAC) method^10^. Given the extreme scale in size and high complexity of the genome structure, none of these single-cell whole genome sequencing methods has revealed genomic details in single cells with complete satisfaction. To date, reports of single-cell whole genome studies have been carried out by employing only one of these three methods, thus there is an urgent need to comparatively evaluate all of the methodologies to guide future research. Recently, de Bourcy *et al.* compared the performance of three single cell sequencing methods (MALBAC, NEB-WGA and MDA) using a bacteria genome^11^. In their study, the comparison was between single-cell MDA in microfluidics, single-cell MDA in tubes, single-cell NEB-WGA in tubes and single-cell MALBAC in tubes. Their results showed that the product using small-volume microfluidics has a higher mapping rate. However, no such studies have been reported for mammalian cells. The goal of this study is therefore to characterize the amplification uniformity and biases for WGA4, MDA and MALBAC based sequencing at shallow sequencing depth using neurons as the model system.

In the human brain, the 85 billion individual neurons^12^,^13^ show remarkable diversity in their maturation, morphology, electrophysiological properties, and inter-neuronal connectivity. Somatic variations of the genome and epigenome, including chromosome instability, aneuploidy (rarely polyploidy), mosaic sub-chromosomal rearrangements, and changes in epigenetic modifications, contribute to the creation of neuronal diversity^14^. Thus, neurons are a suitable system to study single-cell genome diversity. In this study, after the quality of single-neuron genome sequencing was confirmed by comparison to the results of traditional sequencing studies (using genomic DNA from ≈ 2 million neurons of the same rat, referred to as bulk cells in this study), we quantitatively analyzed 19 neurons amplified by the WGA4, MDA and MALBAC techniques with an emphasis on the following questions: 1) Is there amplification bias among different genomic regions, and can the bias issue be addressed? 2) How reproducible are these three whole genome amplification methods? 3) What are the major advantages for each of the three single-cell whole genome amplification methods? Our results demonstrated that single-cell genome sequencing results using either the MALBAC or WGA4 method are highly reproducible and have a high success rate, chromosome-level and sub-chromosomal level CNVs among individual neurons can be detected.

## Results

### Experiment design

**Figure 1:**
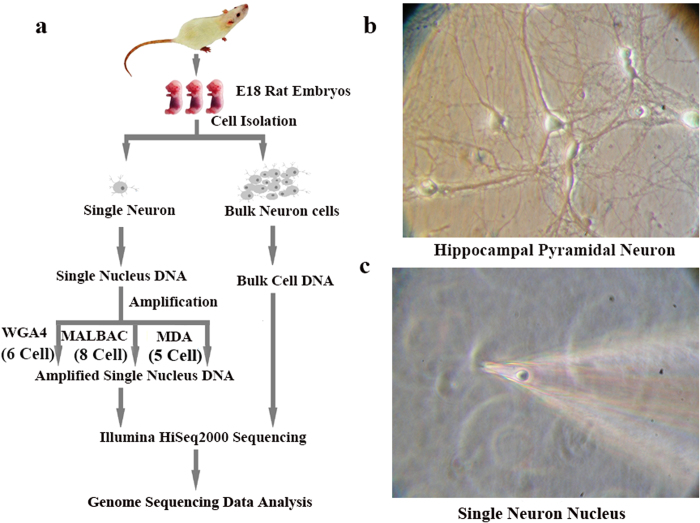
Experimental design. (**a**) Flow chart of bulk-neuron and single-neuron genomic isolation, DNA sequencing sample preparation, sequencing, and data processing. (**b,c**) Hippocampal neuronal culture and isolation of the nucleus from individual neurons. The nucleus was extracted directly from cultured neurons through a glass micropipette on an electrophysiological recording system. A typical isolated nucleus in the micropipette is shown (**c**).

The general strategies that were used in sample preparation, DNA sequencing, and data analysis are summarized in Fig. 1a. Hippocampal neurons were prepared from individual E18 rat embryos and cultured in neurobasal medium as described previously^15^,^16^. Nuclei of individual hippocampal neurons were collected using a glass micropipette (Fig. 1b,c)^17^,^18^ and transferred directly to 200-μL PCR tubes. We collected two batches of single neuron cells for our experiments. The first batch was collected in November 2012 and 11 cells were sequenced in March 2013. The second batch was collected in December 2014 and 8 more cells were sequenced in January 2015 (Supplementary Table S1). The single neuron nucleus was subjected to whole genome amplification using one of three methods (8 nuclei by MALBAC, 5 nuclei by MDA and 6 nuclei by WGA4). At the same time, genomic DNA was also isolated from ≈ 2 million cultured neurons (bulk cells). Sequencing libraries were constructed following the Illumina standard protocol and sequenced by an Illumina HiSeq 2000. On average, there were 29.4 ± 4.8 million clean reads per sample. The data were mapped to the rat reference genome (rnt5) using Bowtie2^19^. Using the sequencing results from bulk cells as the benchmarks, single-cell genome sequencing results were analyzed for genome coverage, GC-bias, reproducibility, and CNVs^20^.

### GC-bias

**Figure 2:**
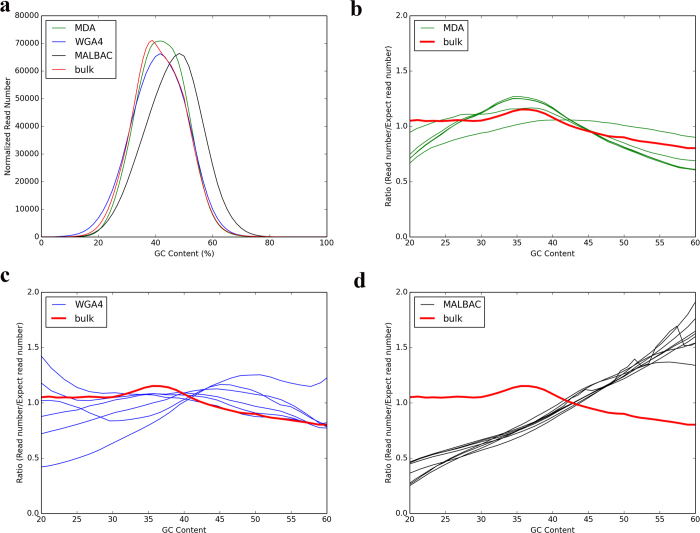
GC content analysis. (**a**) The GC-composition of four samples (bulk-cell sample and DNA amplified by three different single-cell amplification methods). The y-axis is the frequency of normalized reads with different GC contents. The total reads of each sample is normalized to 10 million. The average GC content of the rat reference genome is 41.9% (dashed line). The average GC content of MDA, WGA4, MALBAC, and unamplified samples are 43.4%, 41.6%, 46.6%, and 41.4%, respectively. (**b,c**) The GC-bias plot. The relative coverage (y-axis) represents the ratio between the coverage of a sample and the coverage predicted by the reference genome. A relative coverage of 1 indicates no bias. A relative coverage above 1 or below 1 indicates higher or lower coverage than that expected, respectively. The results from the bulk-cell sample (red line) are plotted as a benchmark.

GC-bias is a parameter that is used to quantitatively evaluate whether there is a correlation between the observed coverage of a specific genomic region and its GC content. The presence of GC-bias can complicate data analysis, including copy number estimation. In single-cell whole genome sequencing studies, GC-bias may result from either the whole genome amplification step or the sequencing step^11^,^21^,^22^,^23^,^24^. In our study, before analyzing individual cells, the sequencing results of DNA from bulk cells were used as the benchmark to quantitatively evaluate the sequencing platform because this sample was sequenced without the need for genome amplification. The average GC content of bulk-cell genomic DNA sequencing data is 41.4%, which is 0.5% less than the GC content of the reference genome (41.9%) (Fig. 2a). Previous studies suggested that the GC-rich regions are prone to low coverage with the Illumina HiSeq 2000 platform^25^. Our results indicated that there is a very low level of GC-bias when sequencing the rat genome using the Illumina HiSeq 2000 platform.

Because of low level of GC-bias at the sequencing step on the Illumina HiSeq 2000 platform, we mainly focused on the GC-bias during the whole genome amplification step. The average GC content (41.6%) of the DNA samples amplified from a single cell using the WGA4 method is very close to that of the reference genome (41.9%), with a difference of ≈ 0.3% (Fig. 2a). The average GC content (43.4%) of the DNA samples amplified from a single cell using the MDA method is ≈ 1.5% greater than that of reference genome (41.9%). However, the average GC content (46.6%) of DNA prepared by the MALBAC method is ≈ 4.7% greater than that of reference genome, indicating some degree of preference for GC-rich regions during the amplification process for MALBAC based amplification. After examining the overall GC-bias, we then analyzed the relationship between the observed reads and the GC content of individual genomic regions (Fig. 2b–d and Supplementary Fig. S1). The majority of rat genomic sequences (75%) have a GC content of 20–60%. Using the sequencing results from bulk cells, the plot of the relative coverage at various genomic regions versus the GC content (20–60%) is almost uniform with a very small bias toward regions having ≈ 35–40% GC. For single-cell genomes amplified using the WGA4 method, a significant level of cell-to-cell variation exists at the low-GC-content regions (<30% GC content). MALBAC has a clear preference for high-GC-content regions relative to the low-GC-content regions. Genomic regions with a high-GC-content tended to be over-amplified when the MALBAC method was used. A similar GC-bias was also noticed when MALBAC was applied to characterize human cancer cell lines^10^. The five MDA cells displayed a similar trend with bulk cell samples, which suggests that the MDA method has a low level of GC bias during the amplification step.

### Reproducibility

**Figure 3:**
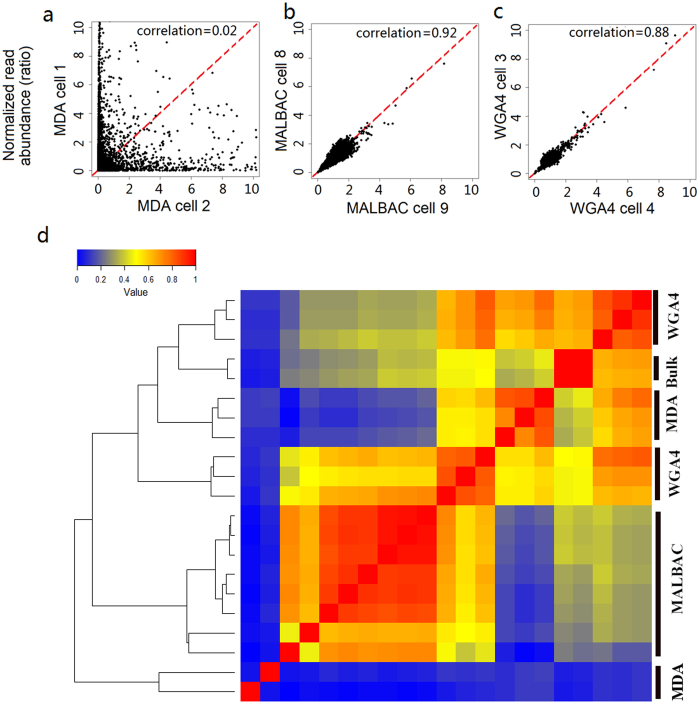
Reproducibility of different whole genome amplification methods. (**a-c**) For each bin that is 500 kb in size, the normalized read abundance of one cell is the x-axis value, and the normalized read abundance of another cell is the y-axis value. The combination of these values will give one dot on the plot for a particular bin. There are 5,797 bins in total. The 5,797 dots are plotted to show the reproducibility of the single-cell genome amplification methods. A narrow distribution of dots along the y = x (red line) indicates good correlation between the two cells. See Supplementary Fig. S2 for all cells used in this study and Supplementary Fig. S3 for results using 200-kb bin size. (**d**) Hierarchical clustering is performed on the correlation of each of the single cells used in this study and the bulk-cell sample.

Because each cell has only a tiny amount of DNA, which is typically at the level of picograms, the success of each experiment highly depends on the quality of the sample, the skills of operators, and potential contamination in the lab. To extract useful information from single-cell genomic studies, one of the key issues to be addressed is to distinguish between true biological cell-to-cell variation and non-specific experimental noise or errors. Thus, the next question addressed in our study was the cell-to-cell genome amplification reproducibility. To evaluate the reproducibility of the three whole genome amplification methods (WGA4, MDA and MALBAC), the rat reference genome was partitioned into 500 kb-sized bins, yielding 5,797 bins in total. After calculating the reads mapped to each bin, we then compared the number of normalized reads of each bin between two representative cells amplified using the same method or amplified using two different methods (Fig. 3a–c and Supplementary Fig. S2 and S3). Our data show that amplifications by both MALBAC and WGA4 are highly reproducible, with a correlation coefficient >0.9 among cells amplified using the MALBAC method and close to 0.9 among cells amplified using the WGA4 method. Such high correlation coefficients suggests that single-cell genome amplification by either the MALBAC or WGA4 method is highly reproducible and that the information extracted from single-cell genomic studies can be used to analyze cell-to-cell genomic diversity. Consistent with a high level of cell-to-cell variation reported in literature for studies using MDA as the amplification method^26^,^27^,^28^, the cell-to-cell correlation of MDA is also very low in our studies.

We also calculated the correlation coefficient matrix between any two cells among the 19 single cells and their correlation with the sequencing results from bulk cells (Supplementary Fig. S4). Hierarchical clustering of the correlation coefficient matrix shows that single cells from MALBAC are clustered together, while single cells from WGA4 are grouped into two clusters (Fig. 3d). Two single cells from MDA display almost no correlation with any other cells. Cells that were amplified using different methods display poor correlation, indicating that each method has its own built-in pattern of biases. Thus, single-cell genomes that are amplified using different methods may not be suitable for comparative studies. Because of their high reproducibility, very useful information might be extracted from single-cell genomic studies using either the WGA4 or MALBAC method once proper GC-bias and other built-in patterns of bias are considered.

### Genome coverage uniformity

**Figure 4:**
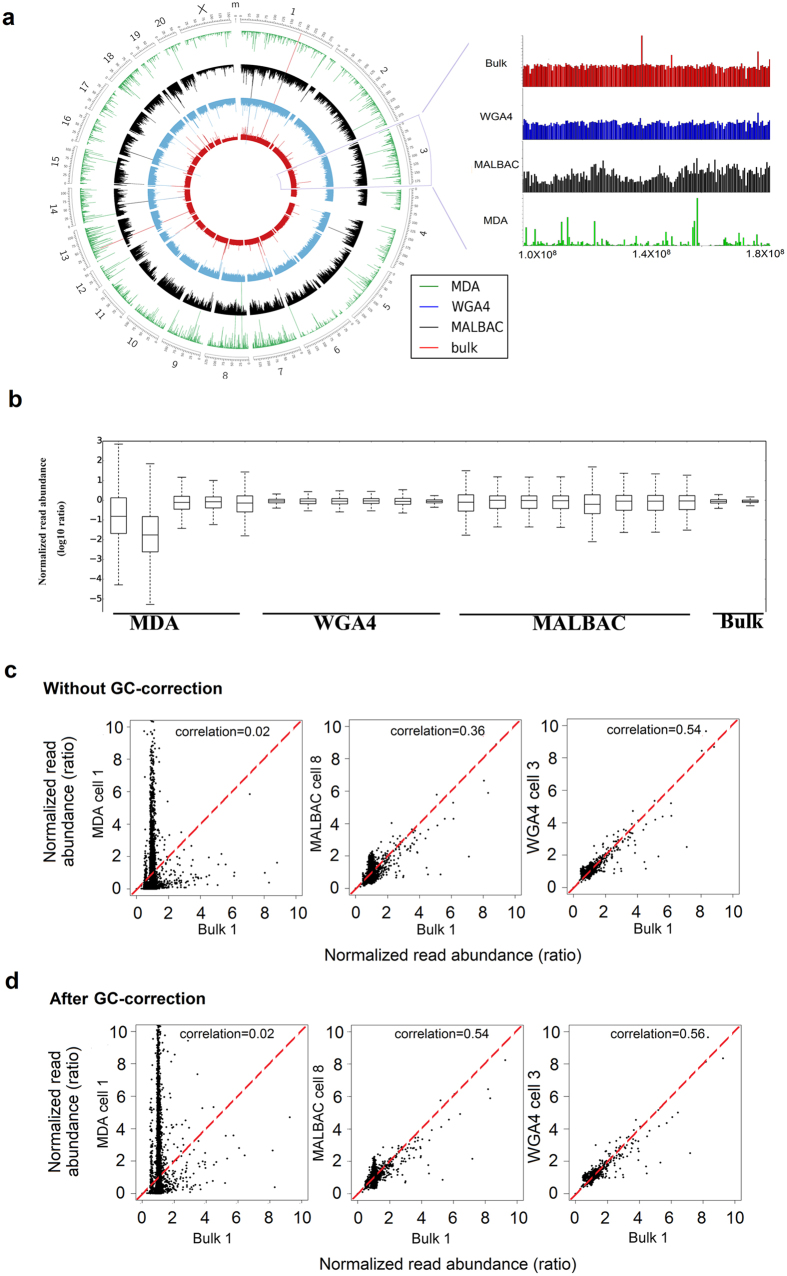
Read abundance. (**a**) Read abundance distribution across the genome from three single-cell amplification methods and a bulk-cell sample (left) and a magnified plot of part of chromosome 3 (right). The five circles from outside to inside are the chromosome position index, read abundance of MALBAC (black), read abundance of WGA4 (blue), and read abundance of the bulk-cell sample (red). Each bar represents the total reads in a 500 kb bin, and all 5,797 bins are plotted. (**b**) Box plot representing the normalized reads in 5,797 bins in log_10_ scale. x-axis are all 19 single cells and bulk sample. y-axis are the average, 25th (Q1), 50th, and 75th (Q3) percentiles of normalized reads for each sample. (**c,d**) Correlation between the three single-cell amplification methods and the bulk-cell sample without GC-correction (**c**) and with GC correction (**d**) in autosome region. The normalized read abundance in a bin of one sample is the x-value and the normalized read abundance of another sample is the y-value. The combination of these two values will give one dot on the plot for a particular bin. A narrow distribution of dots along the y = x (red line) indicates good correlation between a single-cell method and the bulk-cell sample. For each method, one particular cell is chosen for this plot. Supplementary Fig. S4 illustrates the results for all of the other cells.

The genome coverage uniformity represents the evenness of sequence read distribution over the entire genome. Among the three single-cell genome amplification methods examined in this study, samples amplified using the WGA4 method showed the smallest bin-to-bin variation in read abundance (Fig. 4a), which is also consistent with the GC-bias results shown in Fig. 2.

To quantitatively measure the bin-to-bin variation, a box-plot was prepared for each of the cells used in our studies (Fig. 4b). Box plots characterize a sample using the 25th (Q1), 50th, and 75th (Q3) percentiles and the interquartile range (IQR = Q3-Q1). It covers the central 50% of the data. Quartiles are insensitive to outliers and preserve information about the center and spread. Consequently, they are preferred over the mean and standard deviation for population distributions. Among the three single-cell amplification methods, the IQR of the WGA4 method is the smallest, indicating that WGA4 has the least read fluctuation among the bins and the best performance with respect to coverage uniformity. We also plotted the normalized single-cell reads of each bin against results from bulk cells (Fig. 4c). The WGA4 method gives the best correlation (correlation efficiency 0.56) with bulk-cell samples. Random fragmentation of genomic DNA to around 300 bp in the WGA4 method may be responsible for the evenness of the whole genome amplification. Because MALBAC has a GC-bias toward higher GC content (Fig. 3), the correlation between MALBAC and bulk-cell samples is poor. Therefore, we applied a GC-correction, locally weighted scatterplot smoothing (LOWESS) algorithm^29^, to correct the GC-biases in all three single-cell methods and recalculated the correlation (Fig. 4d **and** Supplementary Fig. S5a**, b**). The correlation between MALBAC and bulk-cell samples was significantly improved, from 0.36 to 0.53. There is a slight change in the correlation after GC-correction in the WGA4-amplified samples (from 0.54 to 0.56). However, the sequencing reads by MDA are too random to be correlated and corrected (0.02, no correlations and no improvement after GC-correction). In our hands, the WGA4 method presents superior genome coverage uniformity and correlation with the bulk-cell sample. When single-cell genomes are amplified by MALBAC, after GC-correction, high-quality data can also be obtained.

### MAPD metric to determine the detection limit for CNVs

**Figure 5:**
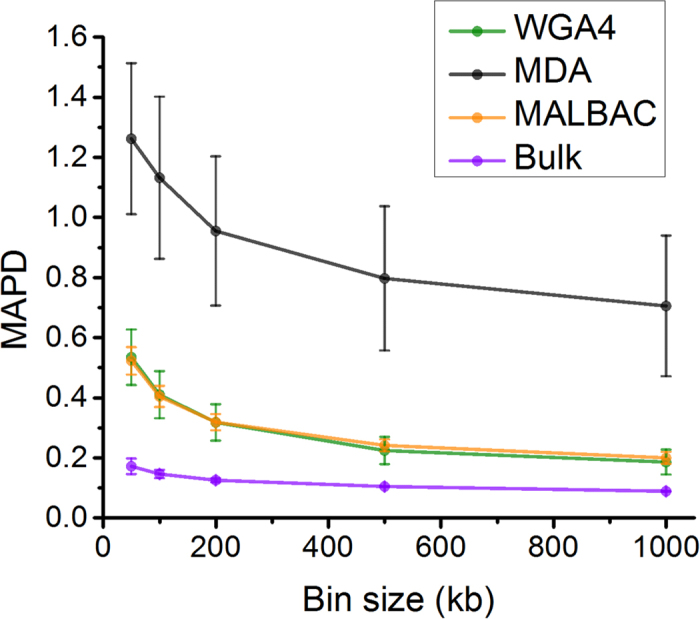
MAPD metric to determine the detection limit of CNVs. The MAPD is calculated in various bin size from 50 kb to 1 Mb. The error bar is standard error.

In order to quantify the amplification biases and noise, we adapted a QC metric, the median absolute pairwise difference (MAPD) algorithm (Affymetrix, 2008)^30^,^31^. MAPD is originally designed for microarray data, and is widely used in the Affymetrix microarray. MAPD measures the absolute difference between the log2 copy number ratios of neighboring bins and then calculates the median across all bins. Larger MAPD values indicate greater noise. For example, at the 500 kb bin size, unamplified bulk cell samples display the lowest MAPD score (mean value: 0.10 ± 0.001), as expected. The WGA4 and MALBAC single-cell samples have similar MAPD scores (mean value: 0.22 ± 0.004 and 0.24 ± 0.002, respectively), both of which are lower than MAPD scores for single-cell samples amplified using the MDA method (mean value: 0.79 ± 0.23). We have also calculated the MAPD scores, while the genomic bin sizes were modulated from 50 kb to 1 Mb. This practice may allow us to further optimize the data analysis and enhance our chances to detect CNVs form single-cell whole genome sequencing information (Fig. 5). MAPD scores decreased with large bin size for all three methods, suggesting less noise with larger bin size. Although single-cell whole genome sequencing always gave higher MAPD scores than bulk sample^7^, this can be partially compensated by increasing the bin size, at the cost of CNV resolution. We chose a MAPD score of 0.45 as the cutoff as suggested by Cai *et al.*^7^. Single-cell samples with MAPD score of >0.45 are considered to be acceptable for CNV analysis. Therefore, single-cell MALBAC and WGA4 methods can reliably detect CNVs using a bin size of 100 kb (Fig. 5). The copy number profiling in MDA single cells are not suitable for small CNV studies less than 1 Mb in size, because the MAPD score is as high as 0.7 even at 1 Mb bin size.

### Detecting copy number variations

**Figure 6:**
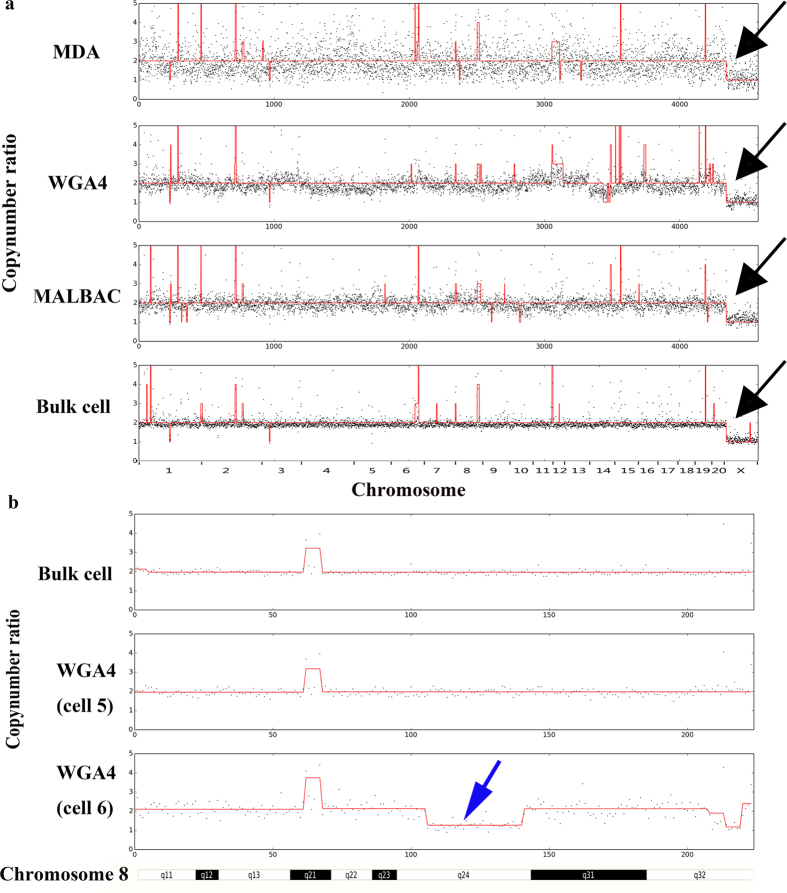
Copy number determination. (**a**) The copy numbers predicted for three single cells and the bulk-cell sample after GC-correction. The black dots are the normalized abundance of reads. The red line is the predicted copy number. (**b**) Mosaic CNV is detected in rat neuron chromosome 8. The single-neuron WGA4 cell 6 has a sub-chromosomal deletion in chromosome 8 that is approximately 20.4 Mb in size.

It has long been accepted that all neurons in a brain share the same genome. However, recent evidence suggests that individual neurons could have non-identical genomes because of aneuploidy, active retrotransposons, and other DNA content variations^32^,^33^,^34^,^35^. We applied the algorithm from Navin *et al.* to call CNVs in neurons^29^. In contrast to using fixed intervals to calculate copy number, we used bins of variable length, while having uniform expected unique read counts. The variable length bin method has been proved to increase the ability to detect CNVs in previous studies^29^. We also used the LOWESS algorithm^25^, which corrects for GC content bias. As shown in Fig. 6a, sequencing results from both the bulk-cell sample and the single neurons amplified by the MALBAC, WGA4, or MDA methods all showed one copy of the X chromosome. These results suggest that all three single-cell whole genome amplification methods can reveal chromosome level CNVs. Fig. 6a shows an example of the CNV detection data quality. At the 500 kb bin size resolution, the copy number patterns of most autosomal chromosome regions (Fig. 6a) from both MALBAC and WGA4 are similar to bulk-cell samples, while the MDA method detected less CNVs than the non-amplification method in bulk-cell samples. A recent study suggested that individual neurons may have somatic mosaic CNVs, in particular, aneuploidy as shown in human postmortem brain samples^2^. We examined such a possibility in rats using our single-neuron sequencing results (Supplementary Table S2). As shown in Fig. 6b, a neuron (labeled WGA4 cell 6) displays a 20.5-Mb sub-chromosomal deletion in chromosome 8, which is not detected in another neuron (labeled WGA4 cell 5) or the bulk-cell sample. This result suggests that rat neurons also have mosaic CNVs, which is consistent with findings in human studies^2^.

## Discussion

In this work, using rat embryonic neurons as the model system, we evaluated the GC-bias, reproducibility, uniformity and the ability to detect CNVs in three single cell amplification methods. The MALBAC method displays amplification preference towards high GC content, while the WGA4 and MDA methods display less GC content biases. Single cells amplified by WGA4 and MALBAC have much higher reproducibility and genome coverage uniformity than that of the MDA method. By using the MAPD metric, we found that both the WGA4 and MALBAC methods can detect the chromosome-level and sub-chromosomal level CNVs, while the copy number profiling in MDA single cells was not suitable for small CNV studies.

In our experiments, the second batch of single cells amplified by MDA method had better quality in the coverage and reproducibility than that of the first batch. We investigated the reason why the second batch has better results than the first batch. The first batch of single cells was isolated and stored in −80 degree for 14 weeks before the MDA amplification performed, while individual neurons of the second batch were isolated, shipped and performed MDA amplification without delay. Also, the handling by different technicians who performed the MDA amplification may also contribute to the variation to some degree.

Choosing which method to use for single cell studies depends on the scientific questions we want to ask. In cancer biology, tumors display extensive somatic mutations and chromosome instability. Single-cell sequencing is now applied to assess the clonal structure of intra-tumoral heterogeneity. In tumor metastasis, single-cell genome sequencing is also suitable for addressing the origin of metastasis and being applied clinically to monitor the metastasis by sequencing single circulating tumor cells^21^. A combination of MALBAC and WGA4 can produce a comprehensive profile of genomic variations in tumors. In pre-implantation genetic diagnosis, we need to select embryos that have the greatest chance for a successful pregnancy and are free of monogenic disorders. MALBAC sequencing of a polar body enabled us to accurately detect aneuploidy and SNPs in disease-associated alleles^36^. In neurobiology, neuronal diversity has been increasingly recognized to be mediated by somatic variations in the genome and epigenome, which mainly include chromosome instability, aneuploidy (rarely polyploidy), mosaic subchromosomal rearrangements, and intercellular changes in the epigenetic profile. Our results show that the WGA4 and MALBAC methods can successfully detect aneuploidy and sub-chromosomal level of CNVs, which is consistent with the report from McConnell *et al.*^2^. In the field of microorganism genomics, many bacteria and archaea are difficult to culture, and single-cell sequencing is a powerful tool to profile their genomes. The MALBAC method is very efficient in amplifying high GC-content regions, which will be at a unique position to sequence genomes with high GC content.

Besides issues associated with genome amplification from single cell nuclei, developing new algorithms for sequencing data analysis is another key area to be addressed in the single-cell whole genome sequencing area. Thus far, few bioinformatics tools are specifically developed for single-cell genomics analysis. Each of the three single-cell amplification methods examined in this report has its own built-in pattern of biases. To develop bioinformatics tools better suited for analyzing single cell whole genome sequencing results, these built-in patterns of bias will need to be considered^20^. For example, MALBAC preferentially amplifies the high-GC-content regions, and this preference is highly reproducible. We can partially correct this bias by normalizing the coverage by the GC content. Better algorithms to identify CNVs are also needed, especially algorithms that enable users to systematically modulate a few parameters to define the resolution of the CNV detection limit for the method used in their specific studies.

In addition to improving the single-cell sequencing techniques, obtaining high quality DNA samples from single cells is also vitally important. In this report, we used micromanipulation with a micro glass pipette to isolate individual nucleus for amplification. A single nucleus is better for amplification than the whole cell because it contains fewer enzymes and proteins that may interfere with the amplification, thus reducing the amplification background. Reducing the amplification reaction volume has also been shown to be suitable for improving the fidelity and reducing the amplification bias. Microfluidics^1^ and nanoliter-based^8^ single-cell amplifications were noted to be able to achieve better data quality than in-tube amplification. To eliminate amplification bias, the third-generation sequencing technologies remove the amplification step before sequencing^37^,^38^, therefore obtaining high quality DNA will become an even more important issue if the third-generation sequencing technologies are used for single-cell sequencing studies.

## Conclusions

We quantitatively compared the performance of three single-cell whole genome amplification methods using rat hippocampal neurons. Using the bulk-cell sample as the benchmark, we have shown that the single-cell DNA sequencing varies in genome coverage, reproducibility, GC-bias, and coverage uniformity. At a similar level of sequencing depth, MALBAC displays the best genome coverage with excellent reproducibility. WGA4 has the best performance in genome coverage uniformity. Findings from this study will guide the selection of an optimal single-cell genome amplification method according to the specific scientific questions to be addressed.

## Methods

### Primary hippocampal neuron culture and single-neuron nucleus isolation

As described previously^15^, hippocampi dissected from embryonic day 18 Sprague–Dawley rat embryos were digested with papain (0.5 mg/mL in Hank’s balanced salt solution HBSS, 37 °C for 20 minutes), washed, and gently triturated by passing the tissue through a Pasteur pipette with a sterile tip. Neurons were counted and plated onto poly-L-lysine (Sigma, 0.5 mg/mL) pre-coated 60-mm Petri dishes (Becton Dickinson, Bedford, MA) at 2 × 10^6^ per dish to isolate DNA from a population of cells (2 million neurons) or dishes containing five glass coverslips (0.3 × 10^6^ per 60-mm dish) for single-neuron nucleus isolation. To ensure high-quality cell adhesion and growth, coverslips were first incubated in 100% nitric acid overnight, thoroughly washed with five changes of large amounts of de-ionized (DI) water, and stored in 70% ethanol. Coverslips were then flamed, dried, coated with poly-L-lysine (Sigma, 0.5 mg/mL) overnight, and washed three times with sterile DI water again before being incubated in plating medium for cell plating. The plating medium is 1× Minimum Essential Media (MEM, Cellgro) containing 10% fetal bovine serum, 5% horse serum (HS), 31 mg L-cysteine, and 1% penicillin/streptomycin/L-glutamine (P/S/G). Twenty-four hours after plating, the plating medium was replaced by feeding medium (Neurobasal medium from Cellgro supplemented with 1% HS, 2% Gibco B-27, and 1% P/S/G). Thereafter, neurons were fed twice per week with 2 mL feeding medium per dish for 2 weeks until use. Given the important roles played by glial cells in neuron development and synaptogenesis, glial cell growth was suppressed by supplementing feeding medium with 5-flouro-2-deoxyuridine beginning on day *in vitro* (DIV) 5, yet they were not completely eliminated from the culture.

A single neuron nucleus was extracted directly through micromanipulation using a micro glass pipette on an electrophysiological recording system. The micro glass pipettes were made on a flaming micropipette puller (Model P-97, Sutter Instrument) by pulling capillary glass tubing (Model G85150T-3, Warner Instruments). The flaming temperature and pulling velocity were adjusted accordingly to generate micropipettes with tip diameters ranging between 5 and 10 μm. The micropipette was then filled with 1× artificial cerebrospinal fluid and installed on the electrophysiological recording system. A micromanipulator (Model MP-225, Sutter Instrument) was employed to control the micropipette to slowly approach the target neurons. Typical hippocampal pyramidal neurons were identified under a 32× objective (numerical aperture, 0.4) with a Zeiss Axiovert 100 microscope. Once in touch with the cell membrane, negative pressure was applied to gently inhale the whole nucleus from the neuron and into the micro glass pipette. The isolated cell (nucleus) in the micro glass pipette was injected into a 200-μl PCR-ready vessel with 3 μl prepared Phosphate buffered saline (PBS, Sigma-Aldrich, Cat no. P5368-10PAK), which was free of DNase, RNase, and pyrogens.

### Whole genome amplification

WGA4 amplification was performed on a single neuron nucleus as described in the Sigma-Aldrich GenomePlex WGA4 kit (Sigma-Aldrich, Cat no. WGA4-10RXN). Briefly, we first lysed the nucleus and removed the proteins by incubating the mixture at 50 °C for 1 hour. The genomic DNA was fragmented for 4 minutes at 99 °C. A set of random primers linked with common adaptors was annealed to the fragmented DNA template at the following series of temperatures: 16 °C for 20 minutes, 24 °C for 20 minutes, 37 °C for 20 minutes, 75 °C for 5 minutes, and 4 °C hold. Then, PCR was performed to amplify the library with an initial denaturation at 95 °C for 3 minutes, and 25 cycles of 94 °C for 30 seconds and 65 °C for 5 minutes. The PCR product was purified using the Qiagen PCR Purification kit. Most DNA in the library is between 200 bp to 400 bp. The single-cell amplification by the MALBAC method was performed by Yikong Genomics based on the methods reported by Zong *et al.*^10^ (http://www.yikongenomics.cn/).

### Sequencing library preparation and sequencing

After single-cell genomic DNA was amplified, the sequencing libraries were constructed by BGI-Shenzhen and sequenced using the Illumina HiSeq 2000 sequencing platform.

### Bioinformatics analysis

#### (A) Read alignment

The total number of reads for each sample ranged from 8 million to 58 million. Because the MALBAC and WGA4 methods added around 30 bp adaptors to each read, we deleted the nucleotide sequences of adaptors and truncated the reads to 60 bp. This truncation was performed for all samples to ensure that all single-cell sequencing data were evaluated using reads of the same length. After filtering for clean reads, the data were mapped to the rat reference genome (rnt5) using Bowtie2 software with the default parameters. Duplicates were removed using SAMtools^39^ and MarkDuplicates from the Picard software suite.

#### (B) GC-bias

To calculate the GC-composition of the reference genome, we divided the reference rat genome into continuous 60 bp windows. The GC content of each window was calculated. The frequency of reads of 1% of the GC-content intervals was counted. To calculate the GC-composition of single-cell samples and the bulk-cell sample, we determined the GC content of each sequencing read, which was used to calculate the relationship between the read distribution frequency and GC content. We normalized the total reads of each sample to 10 million.

Relative coverage is defined as the ratio of the normalized read number of a particular sample to the normalized read number of the reference genome. A relative coverage of 1 indicates that a particular base is covered at the expected average rate. A relative coverage above 1 indicates higher than expected coverage, and a relative coverage below 1 indicates lower than expected coverage. This was used to generate Fig 2.

#### (C) Reproducibility

We divided the rat genome into 500 kb bins. The total bin number is 5,797. We then calculated the ratio between the number of reads of each bin and the average number of reads of all 5,797 bins. To quantitatively evaluate the reproducibility, a plot was generated using the ratio of each of the 5,797 bins from the first single cell as the x-value and the ratio of the same bin from the second single cell as the y-value. The correlation coefficient between the ratios of two single cells was calculated. For perfectly reproducible data, data points should all fall onto the y = x line. Hierarchical clustering was performed using the “hcluster” command in R language.

#### (D) LOWESS model of GC-correction

We employed the LOWESS model to perform GC-correction. First, the GC content and read count was calculated for each bin. Then, a local linear polynomial fit was performed for the GC content and read count. Finally, the regression value of the read count was used to replace the original read count for each bin. The LOWESS model for GC-correction has been systematically studied in previous reports^25^,^29^. We adopted a LOWESS function in the R package for this study.

#### (E) MAPD metrics

For quality control purposes, we need to define a metric that demonstrates whether the single-cell whole genome sequencing will produce data that is useful for copy number analysis. This metric is Median of the Absolute values of all Pairwise Differences (MAPD). The MAPD QC metric was developed by adapting the Affymetrix multiple absolute pairwise differences algorithm (Affymetrix, 2008), and is defined as: *MAPD* = *Median*(*log*_*2*_*CNR*_*i*_ *+* *log*_*2*_*CNR*_*i+1*_), where i stands for individual bins. 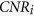 stands for the copy number ratio. Affymetrix recommended a MAPD threshold of 0.40 for CNV calling in microarray. Cai *et al.* recommended a MAPD threshold of 0.45 for all single-cell samples^7^. In our study, we adopted the MAPD threshold of 0.45.

#### (F) Copy number calculation

We used the method proposed by Navin *et al.* to calculate CNV^9^. Copy number was assessed with bins of variable size. We first calculated the number of reads that were mapped to each bin. Then, we performed the GC-correction using the LOWESS model^19^. We used the circular binary segmentation algorithm from an R package (DNA copy) to group adjacent bins into segments under the following settings^40^: *alpha* = *0.02, nperm* = *1000, undo.splits* = *“sdundo”, undo, SD* = *1.0, min.width* = *5*. The copy number of each segment was calculated as the mean read number of bins in the segment divided by the mean read number of bins of all autosomal chromosomes (multiplied by 2). A CNV was called if a given genomic segment met the following criteria: (1) spans at least 4 genomic bins; (2) >2 Mb in size; (3) Segment mean is larger than 2 times of median absolute distance of the segment means of the samples. The copy number is shown as the blue line in Fig. 6a. We rounded the copy number to integers. The rounded copy number is shown as the red line in Fig. 6a.

## Additional Information

**How to cite this article**: Ning, L. *et al.* Quantitative assessment of single-cell whole genome amplification methods for detecting copy number variation using hippocampal neurons. *Sci. Rep.* **5**, 11415; doi: 10.1038/srep11415 (2015).

## Supplementary Material

Supplementary Information
