# Supplementary material for: Quantitative assessment of single-cell whole genome amplification methods for detecting copy number variation using hippocampal neurons
Source: Sci Rep. 2015 Jun 19;5:11415. doi: 10.1038/srep11415 (PMC4650676; doi:10.1038/srep11415)
Supplement: Supplementary Information [file srep11415-s1.doc]

**Supplemental Materials**

**Quantitative assessment of single-cell whole-genome amplification methods for detecting copy number variation in hippocampal neurons**

**Running title: Single Cell Sequencing Methods Evaluation**

Luwen Ning^1^**^#^**, Zhoufang Li^1^**^#^**, Guan Wang^2^**^#^**, Wen Hu^3, 4^**^#^**, Qingming Hou^2^, Yin Tong^1^, Meng Zhang^1^, Yao Chen^1^, Li Qin^4^, Xiaoping Chen^4^, Heng-Ye Man^2^*, Pinghua Liu^3^*, Jiankui He^1^*

^1^Department of Biology, South University of Science and Technology of China, Shenzhen 518055, China; Departments of ^2^Biology and ^3^Chemistry, Boston University, Boston, MA 02215, USA; ^4^State Key Laboratory of Respiratory Disease, Guangzhou Institute of Biomedicine and Health, Chinese Academy of Sciences, Guangzhou 510530, China

**Email address for corresponding authors**

***Heng-Ye Man**[hman@bu.edu](mailto:hman@bu.edu) (corresponding author)

***Pinghua Liu**[pinghua@bu.edu](mailto:pinghua@bu.edu) (corresponding author)

***Jiankui He** [hejk@sustc.edu.cn](mailto:hejk@sustc.edu.cn) (corresponding author)

**Sequencing data statistics**

We collected two batches of single neuron cells for our experiments. The first batch is collected in March 2013. The second batch is collected in December 2014. We sequenced 19 single cells in total using whole genome amplification and 2 bulk cell samples. Sequencing was performed in Illumina Hiseq 2000 platform, obtaining 29.4 ± 4.8 million pass-filter reads per sample on average. The sequencing quality varies even using the same method. We kept all cells in the manuscript even some samples have relatively low quality, to help the readers to understand the risk of single cell sequencing.

**Determining the boundary of Bins**

We first divided the rat reference genome (rnt5) into bins with an equal number of simulated reads. To determine bin boundaries, we simulated genome sequence reads by sampling sequences of 60 bp in length at 60 × genome coverage. These sequences were mapped back to the rat reference genome using Bowtie2 with default parameters. The boundaries between bins were set at positions to make each bin with the same number of simulated reads. Copy number is calculated from read density by counting the number of unique reads in each bin.

**Table S1**. The statistics of sequencing data

| Methods | Cell | Rat sample | Reads (M) | Depth* | Coverage^+^ |
| --- | --- | --- | --- | --- | --- |
| MDA  (batch 1) | Cell 1 | Rat embryo 2 | 29.1 | 0.96 | 0.21 |
|  | Cell 2 | Rat embryo 2 | 20.8 | 0.68 | 0.07 |
| MDA  （batch 2） | E1-1 | Rat embryo 3 | 38.3 | 1.17 | 0.37 |
|  | E1-5 | Rat embryo 3 | 39.3 | 1.20 | 0.48 |
|  | E1-6 | Rat embryo 3 | 38.9 | 1.17 | 0.31 |
| WGA4  (batch 1) | Cell 3 | Rat embryo 1 | 8.4 | 0.15 | 0.04 |
|  | Cell 4 | Rat embryo 1 | 8.0 | 0.14 | 0.03 |
|  | Cell 5 | Rat embryo 1 | 27.5 | 0.51 | 0.15 |
|  | Cell 6 | Rat embryo 1 | 8.0 | 0.15 | 0.03 |
| WGA4  (batch 2) | E1-13 | Rat embryo 3 | 10.8 | 0.21 | 0.09 |
|  | E1-14 | Rat embryo 3 | 19.0 | 0.27 | 0.13 |
| MALBAC  (batch 1) | Cell 7 | Rat embryo 2 | 72.3 | 1.30 | 0.24 |
|  | Cell 8 | Rat embryo 2 | 16.9 | 0.31 | 0.14 |
|  | Cell 9 | Rat embryo 2 | 55.3 | 1.01 | 0.26 |
|  | Cell 10 | Rat embryo 2 | 73.6 | 1.34 | 0.28 |
|  | Cell 11 | Rat embryo 2 | 57.8 | 1.00 | 0.16 |
| MALBAC (batch 2) | E1-7 | Rat embryo 3 | 51.0 | 1.62 | 0.39 |
|  | E1-8 | Rat embryo 3 | 30.1 | 0.85 | 0.29 |
|  | E1-9 | Rat embryo 3 | 32.7 | 1.00 | 0.33 |
| BULK | BULK 1 | Rat embryo 2 | 17.8 | 0.55 | 0.36 |
|  | BULK 2 | Rat embryo 2 | 178.8 | 5.47 | 0.86 |

* The total genome size is 2.9 Gb. However, since rat reference genome contains lots of ambiguous nucleotides “N”. If all “N” are removed in the reference genome, the coverage will increase. The depth is calculated only using mapped bases.

^+^This data is based on clean read with adaptor and primer sequences removed.

|  |  |  |  | **WGA4** | **WGA4** | **WGA4** | **WGA4** | **MALBAC** | **MALBAC** | **MALBAC** |
| --- | --- | --- | --- | --- | --- | --- | --- | --- | --- | --- |
| Position | bin | bulk-1 | bulk-2 | cell 3 | cell 4 | cell 5 | cell 6 | cell 8 | cell 9 | cell 10 |
| chr1:147719969-153039724 | 227-234 | 1 | 1 |  |  |  |  |  | 1(228-234) | 1 |
| chr1:212598746-218670380 | 330-340 |  |  |  |  | 1 |  |  |  |  |
| chr2:1-173410319 | 459-470 | 3 | 3 | 7(459-460) | 3 | 3(459-466) | 3(459-467) |  |  |  |
| chr2:173970794-183758117 | 712-720 | 4 | 4 | 4(714-720) | 4(713-720) | 5 | 4(714-720) | 4(714-720) | 4(714-720) | 4(710-720) |
| chr2:209215879-217182384 | 764-773 | 3 | 3 |  | 3(764-776) |  |  |  | 3 |  |
| chr3:21139650-27014788 | 917-924 |  |  |  |  |  | 3 |  |  |  |
| chr3:50884957-53720837 | 966-970 | 1 | 1 |  |  |  |  |  |  |  |
| chr3:77686537-100719718 | 1013-1050 |  |  |  |  |  | 1 |  |  |  |
| chr4:163510873-166324464 | 1359-1363 |  |  |  |  | 1 |  |  |  |  |
| chr4:182802977-194010696 | 1389-1408 |  |  |  |  | 1 |  |  |  |  |
| chr4:234192714-239869199 | 1477-1486 |  |  |  |  | 1 |  |  |  |  |
| chr5:134182320-137217933 | 1719-1723 |  |  | 1 |  | 1 |  |  |  |  |
| chr6:29089492-32563473 | 1825-1830 |  |  |  |  |  |  |  |  | 1 |
| chr6:96734054--100955185 | 1942-1948 |  |  |  |  |  |  |  |  | 1 |
| chr6:116032687-137018906 | 1975-2011 |  |  |  |  |  |  |  |  | 1 |
| chr7:1-18668201 | 2043-2066 | 3 | 3 | 3 | 3 | 3 | 3 (2004-2066) |  | 3 |  |
| chr7:18668201-23975189 | 2067-2071 | 8 | 8 | 5 | 5 | 6 | 6 | 5 | 6 | 5 |
| chr8:37223795--42706283 | 2341-2346 | 3 | 3 | 3 | 3(2340-2346) | 3 | 3 |  | 3(2337-2351) |  |
| chr8:63881933-84278183 | 2385-2419 |  |  |  |  |  | 1 |  |  |  |
| chr8:126506078-129648809 | 2493-2498 |  |  |  |  |  | 1 |  |  |  |
| chr9:3-11996085 | 2503-2518 | 4 | 4 | 3 | 3 | 3 | 3 | 3(2503-2522) | 3 | 3(2503-2527) |
| chr11:38097183-43729488 | 2962-2970 |  |  |  | 3 |  |  |  |  |  |
| chr12:1-5790022 | 3056-3060 |  |  | 4 | 4(3056-3061) | 5 | 4(3056-3061) |  |  |  |
| chr13:30862793-37756377 | 3176-3186 |  |  |  |  |  | 1 |  |  |  |
| chr13:81566685-91622932 | 3263-3280 |  |  |  |  | 1 |  |  |  |  |
| chr14:93770536-98821780 | 3487-3491 |  |  | 3 |  |  | 3(3484-3488) |  |  | 3 |
| chr15:31487633-36269413 | 3562-3566 |  |  |  |  | 4 |  |  |  |  |
| chr15:93935557-108994003 | 3665-3690 |  |  |  |  | 1 |  |  |  |  |
| chr16:21564691-40186406 | 3736-3762 |  |  |  |  |  |  |  |  | 3 |
| chr16:40186406-64177924 | 3762-3803 |  |  |  |  |  | 1 |  |  | 3(3776-3785) |
| chr17:13449621-26482251 | 3867-3889 |  |  |  |  |  |  |  | 3 |  |
| chr18:24307817-29181831 | 4040-4048 |  |  |  |  |  |  | 1 |  |  |
| chr19:1-35762508 | 4146-4187 |  |  |  |  |  |  |  | 3 |  |
| chr19:36798847-40765578 | 4190-4193 | 8 | 6 (4190-4194) | 6 (4189-4193) | 7 (4189-4193) | 6 (4188-4193) | 6 (4189-4193) | 6(4189-4193) | 6(4188-4193) |  |
| chr20:3-7691985 | 4251-4258 |  | 3 |  |  |  |  |  | 3(4251-4254) |  |
| chr20:54387956-57387900 | 4340-4344 |  |  |  |  |  |  |  | 3 |  |

**Table S2**. Predicted CNVs in single neurons and bulk cell sample. The first column is the position of CNVs in chromosome. The second column is the bin index. The third to ninth column is the CNVs detected in bulk cell and single cells. The copy number is in red color. We leave this value empty if the copy number is 2. The starting and ending position of a CNV is also provided in bracket if it is not completely overlapped with the bulk cell (in the unit of bin index). The bulk-cell sample has 10 CNVs. Among the 10 CNVs detected from the bulk-cell sample, eight CNVs are also detected in the MALBAC sample. Another two CNVs that are detected in bulk-cells are not observed in the MALBAC sample. The WGA4 sample also has 10 CNVs, and eight of them are also observed in the bulk-cell sample. These comparative studies indicate that single-cell sequencing can reveal not only the information obtained from bulk-cell samples but also other fine details at the single-cell level.

| a | b |
| --- | --- |
| 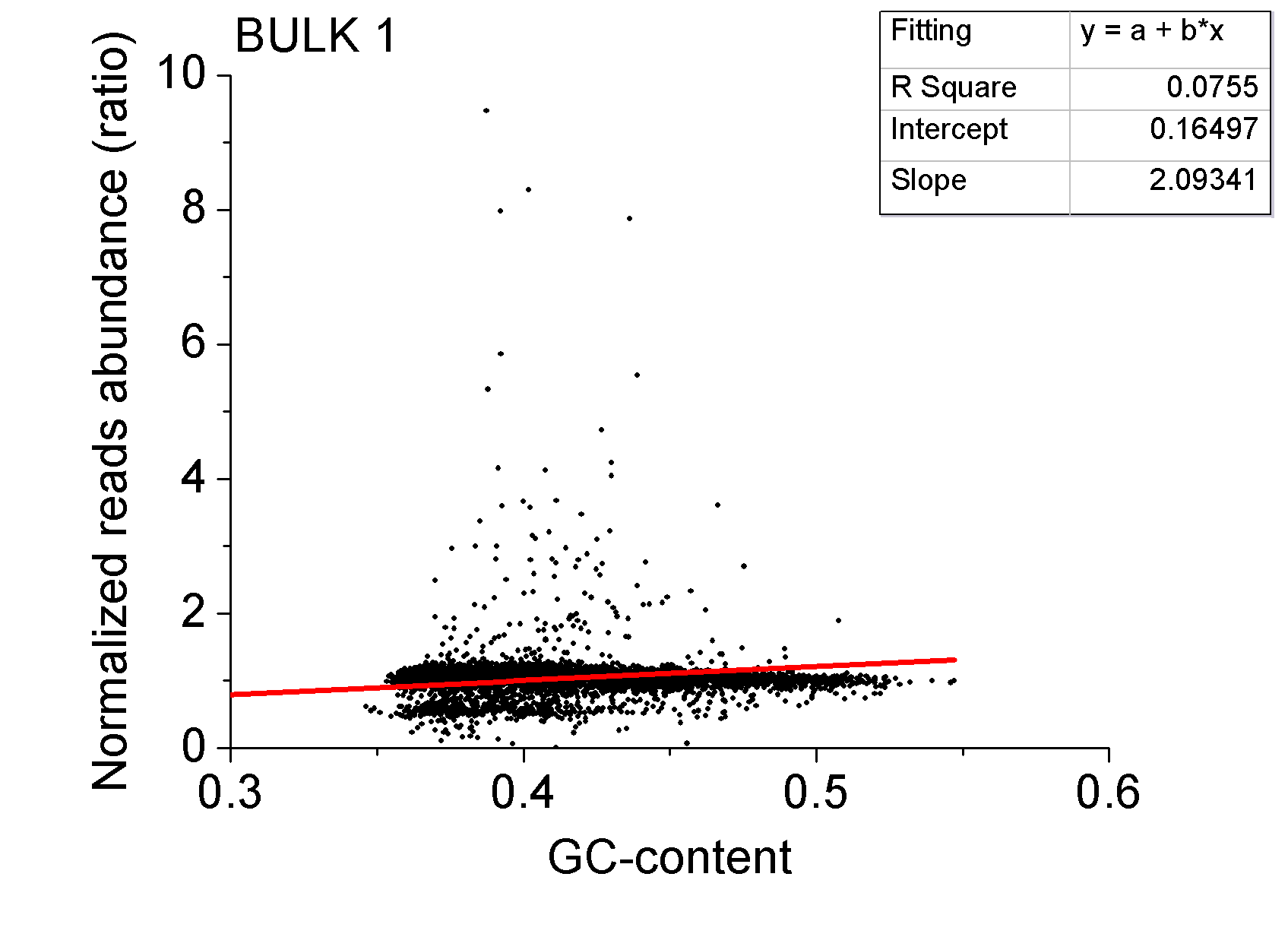 | 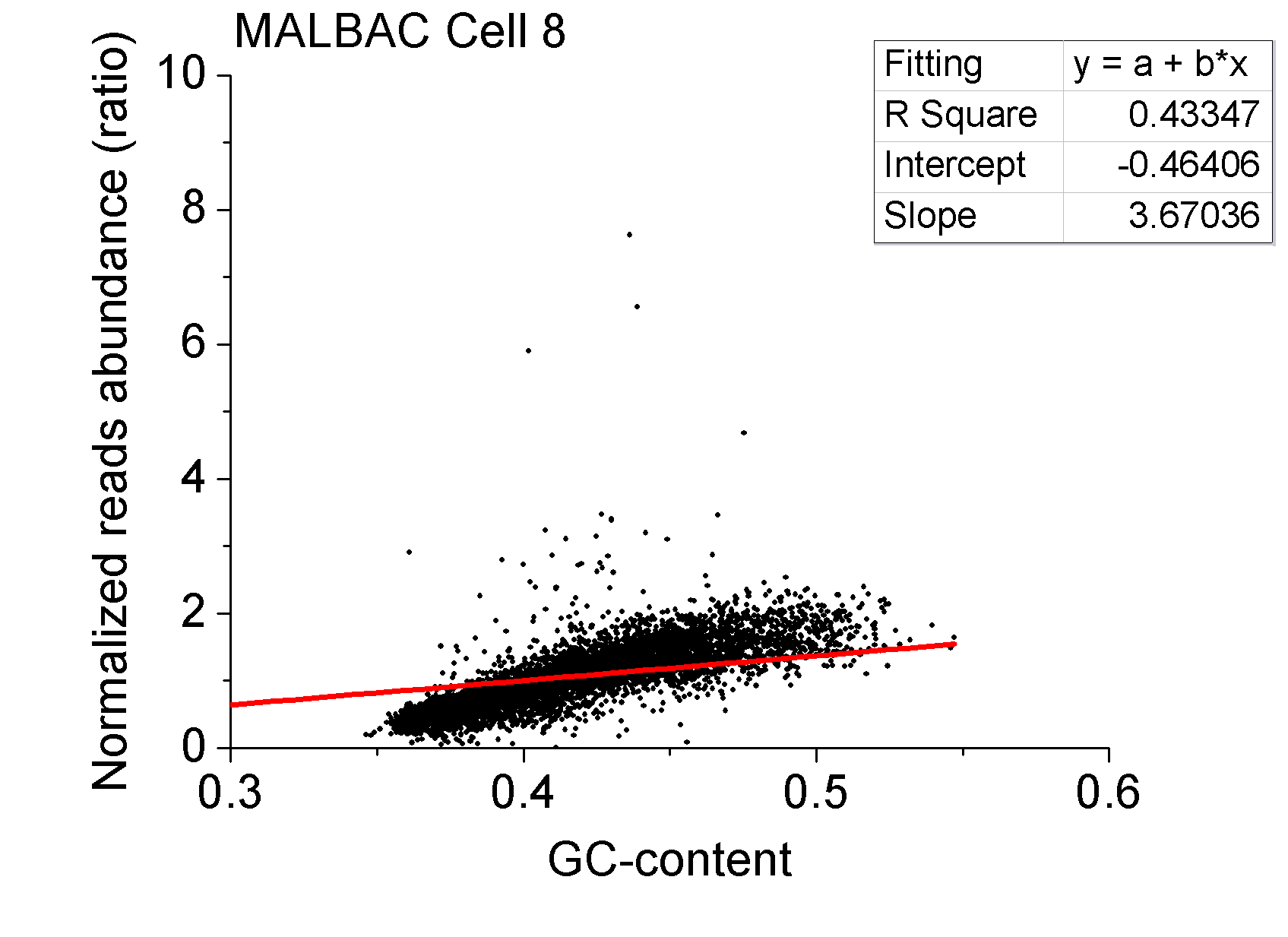 |
| c | d |
| 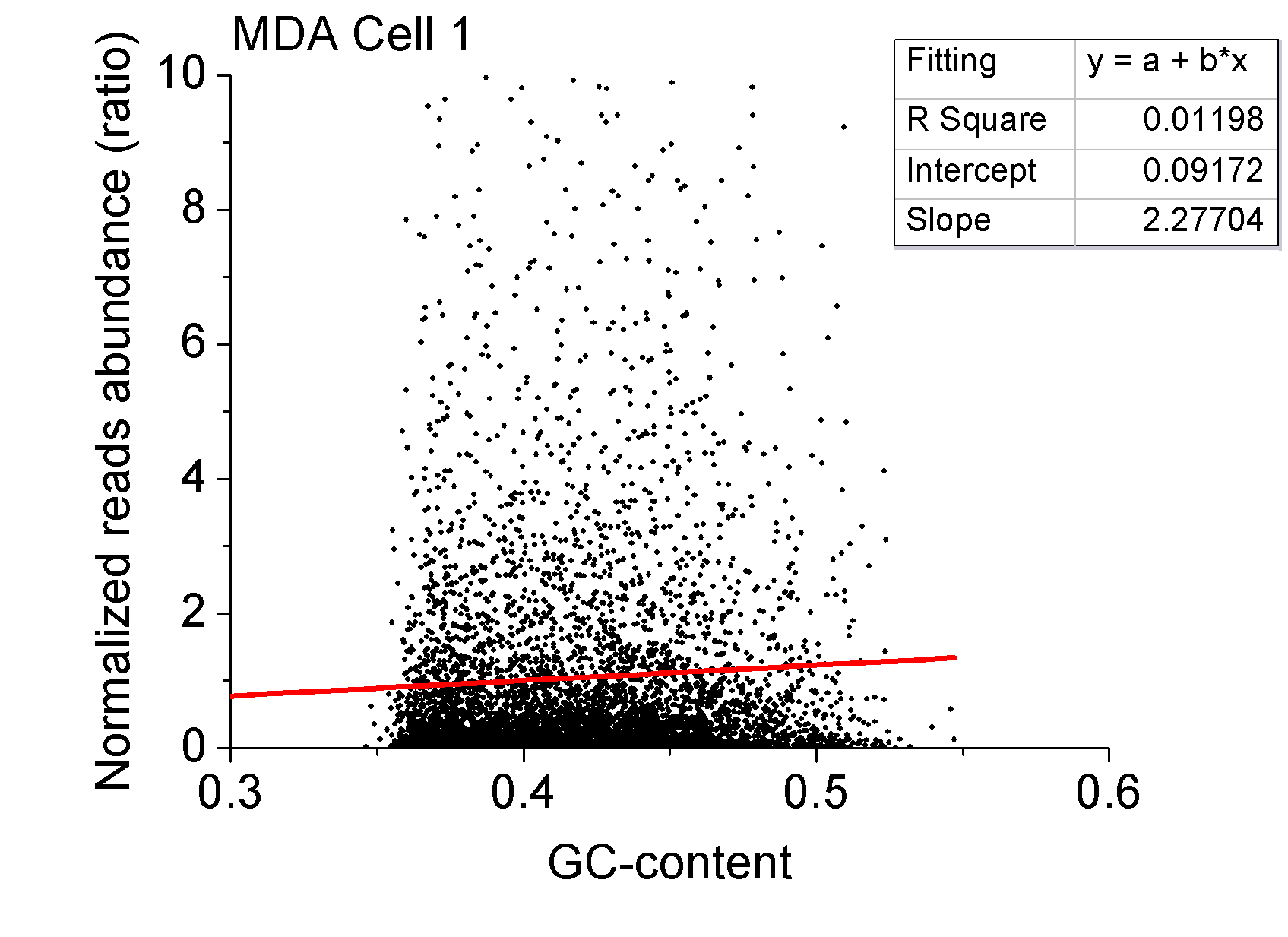 | 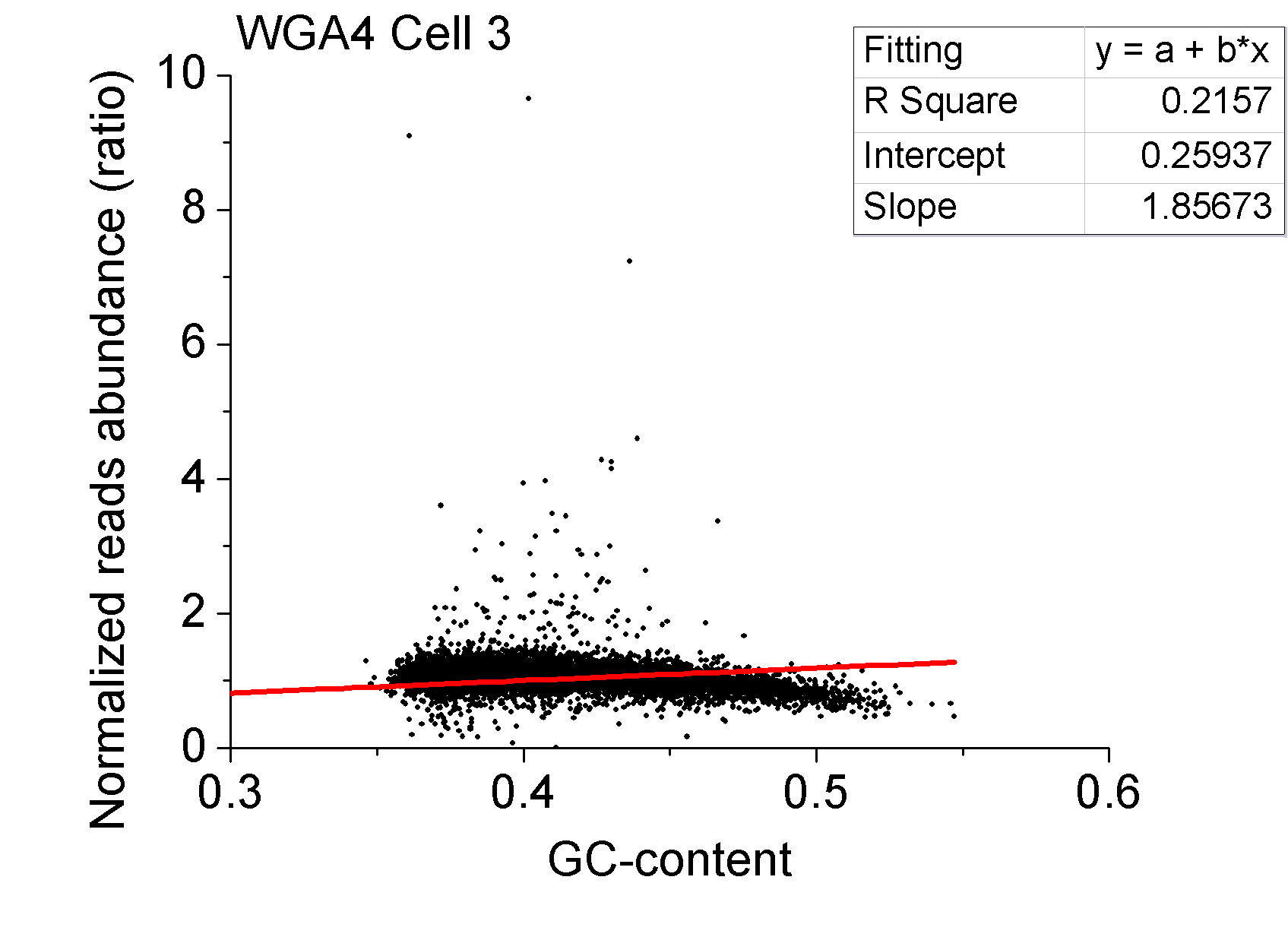 |

**Fig. S1 |** The GC-content bias across the genome. Each data point represents a 500 kb bin. Y axis is the number of reads mapped to a particular bin and x axis is the GC-content of the same region in reference genome. The MALBAC displays a positive correlation between the GC content and the reads abundance (R^2^=0.43), indicating that MALBAC preferentially amplify GC-rich regions. Bulk sample and MDA displays no significant correlation between GC content and read abundance. The linear fitting of WGA4 cell is not significant.

| 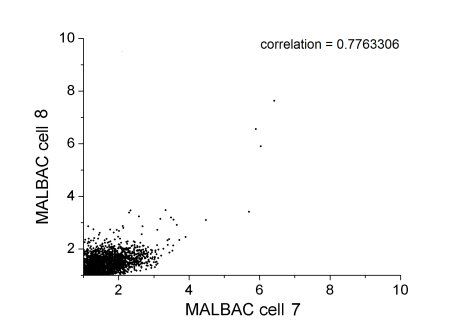 | 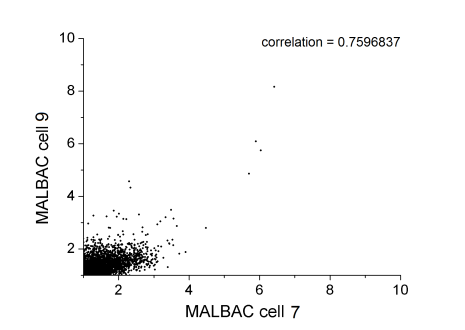 | 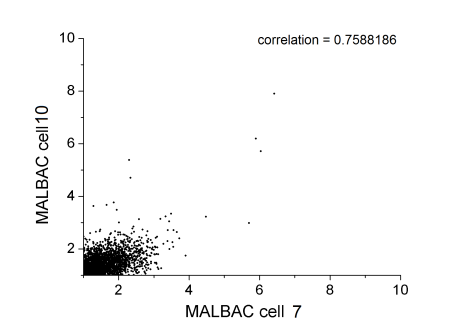 |
| --- | --- | --- |
| 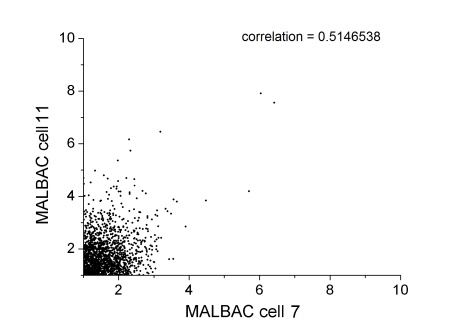 | 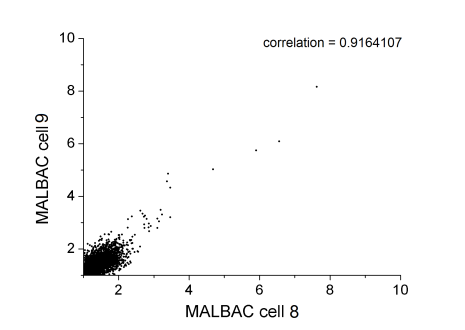 | 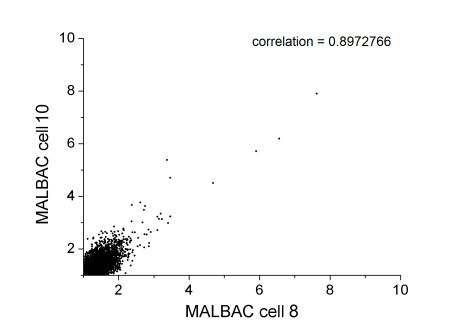 |
| 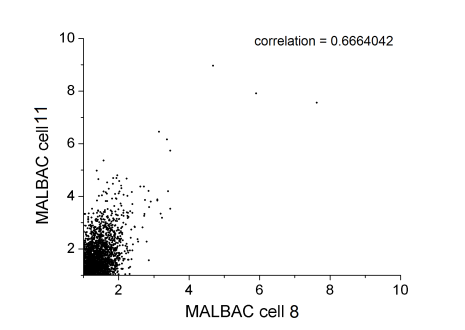 | 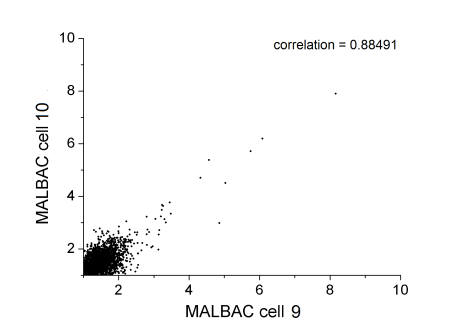 | 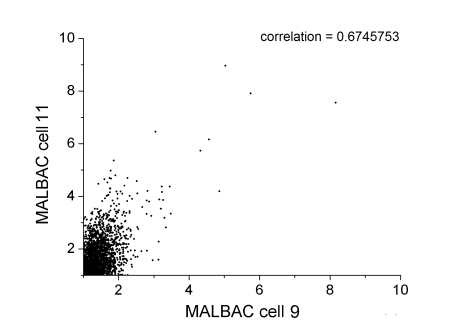 |
| 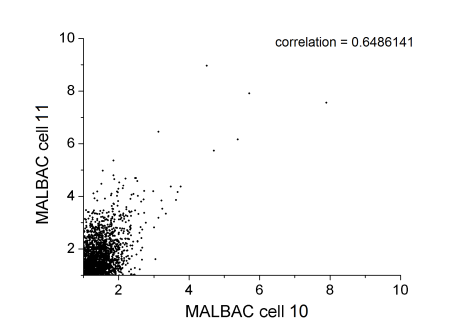 | 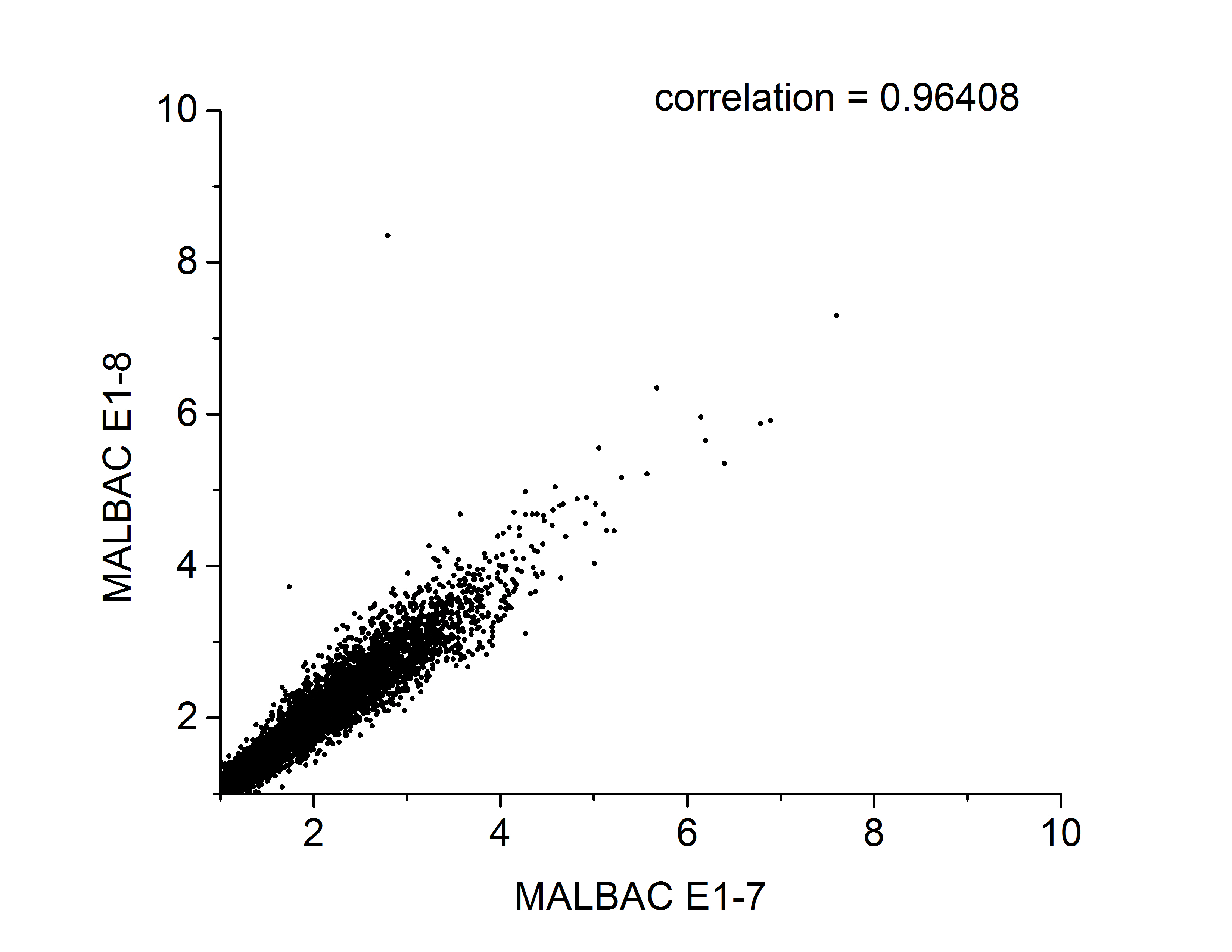 | 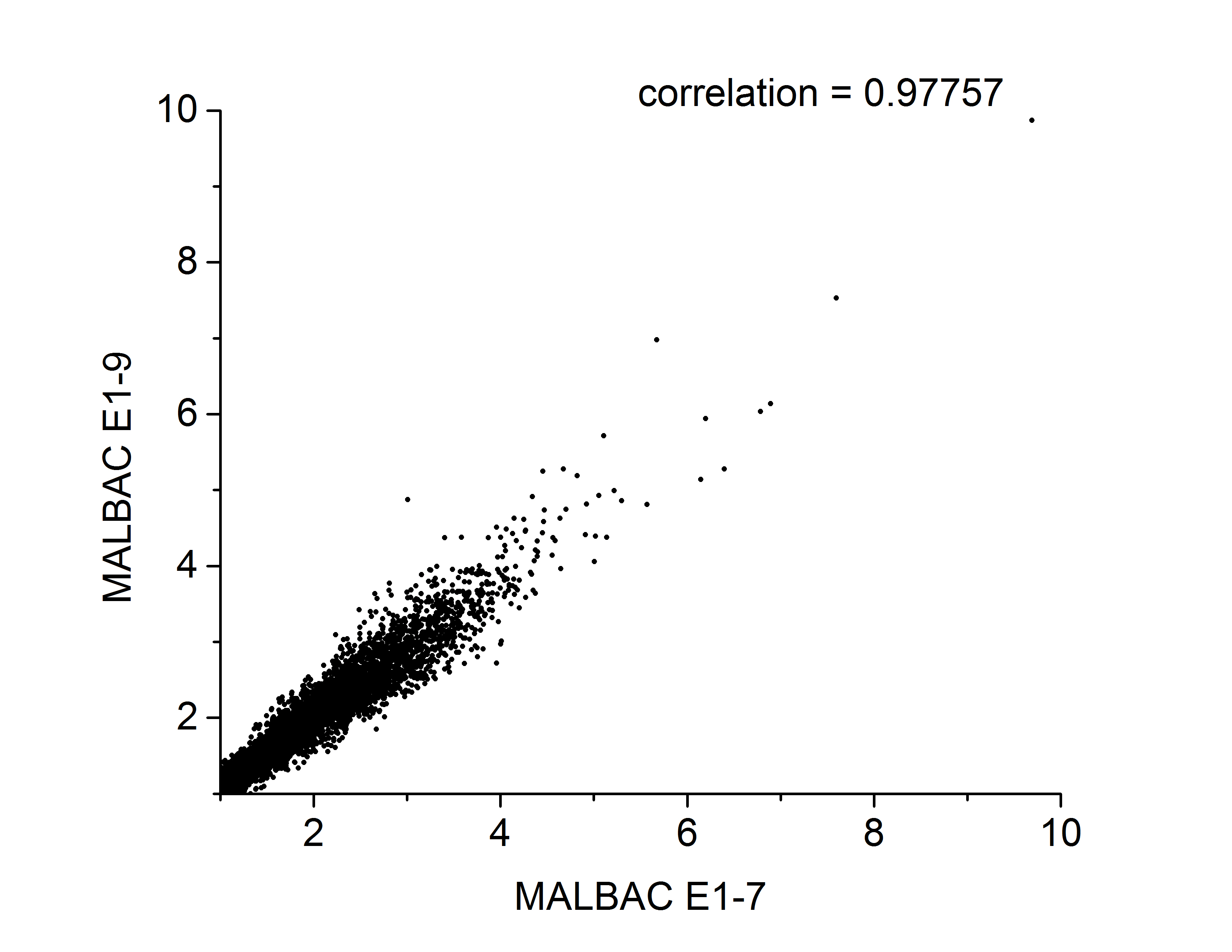 |
| 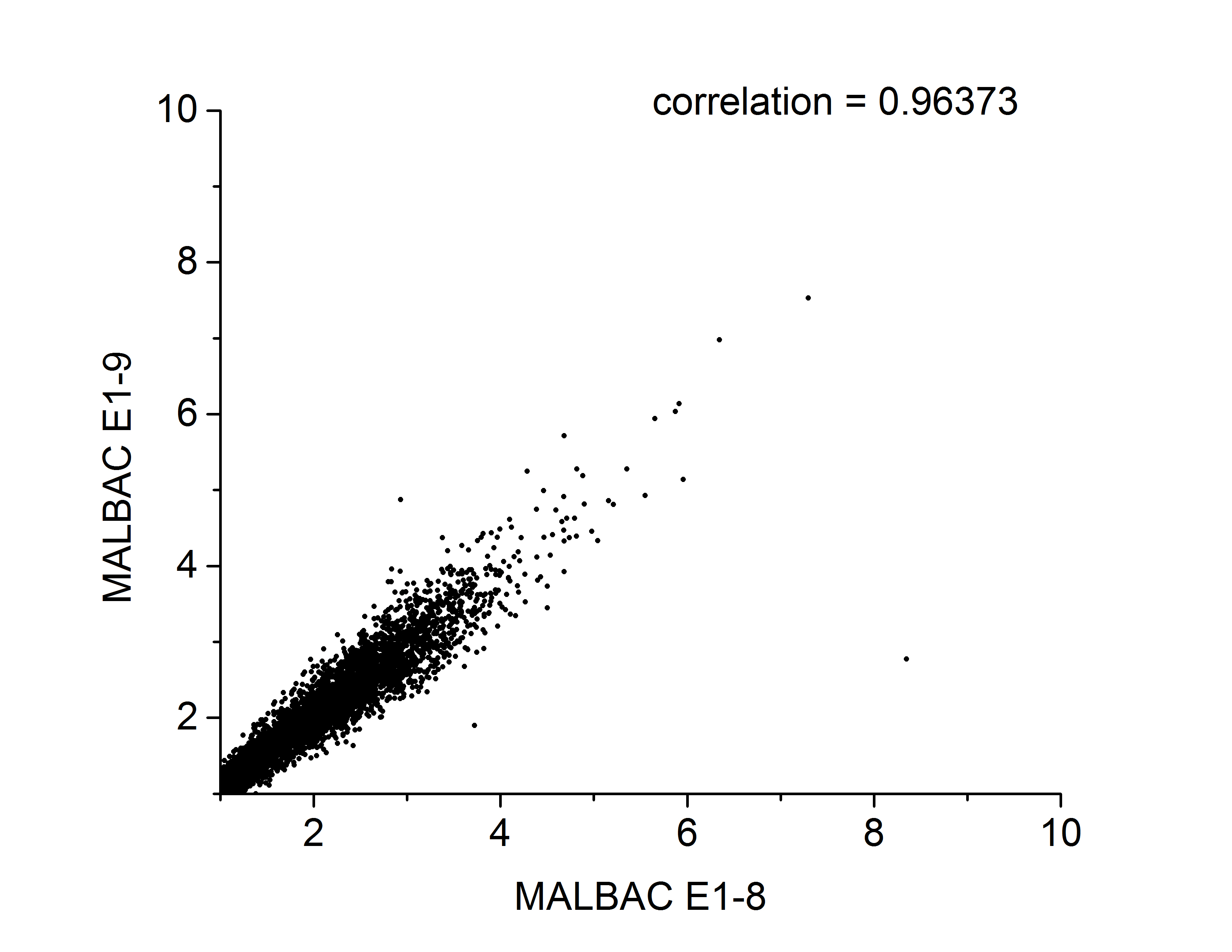 | 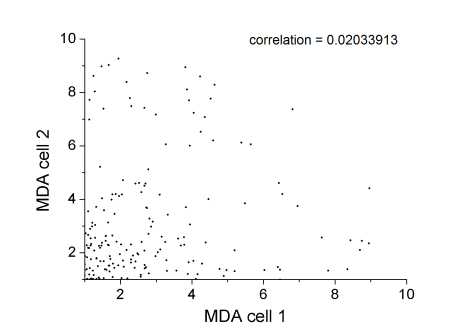 | 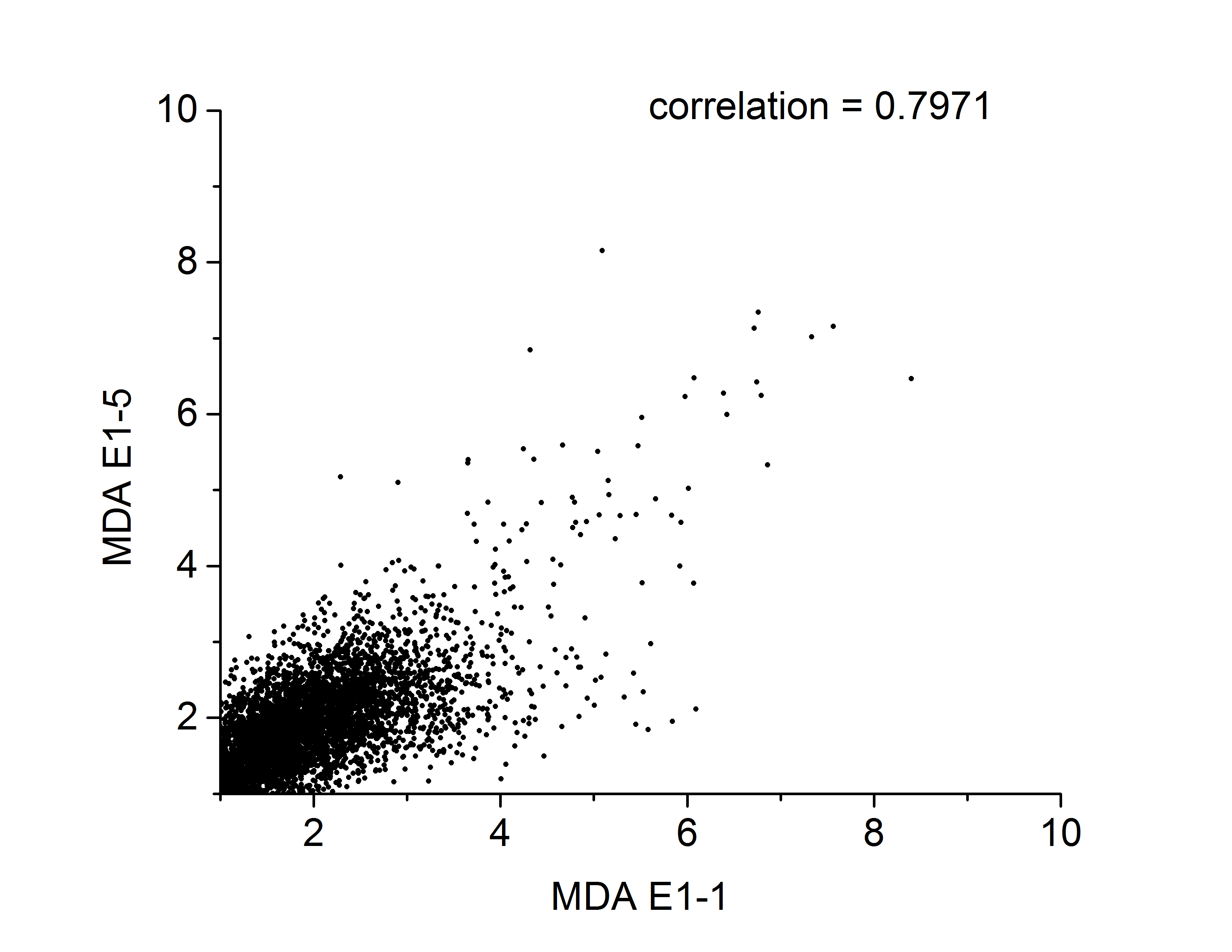 |
| 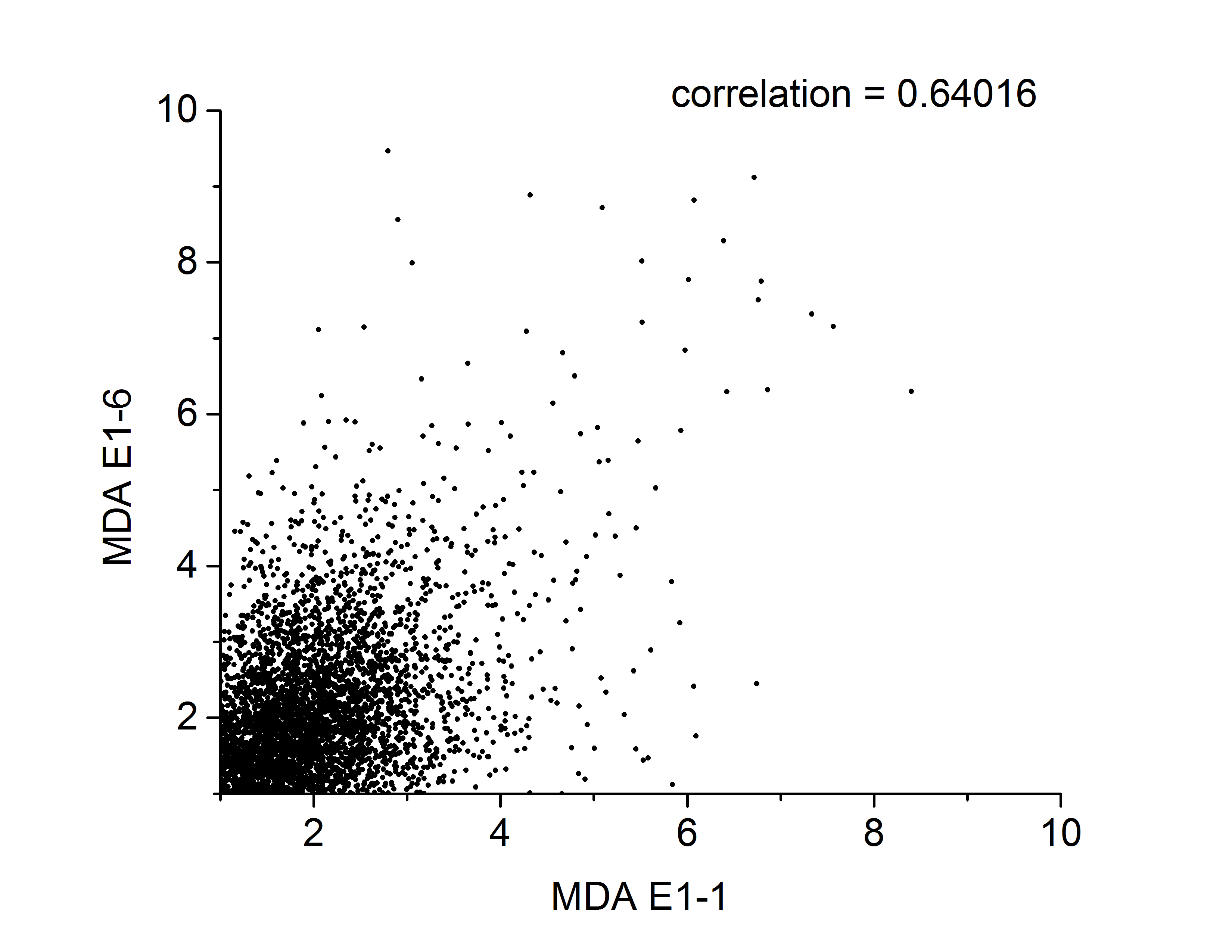 | 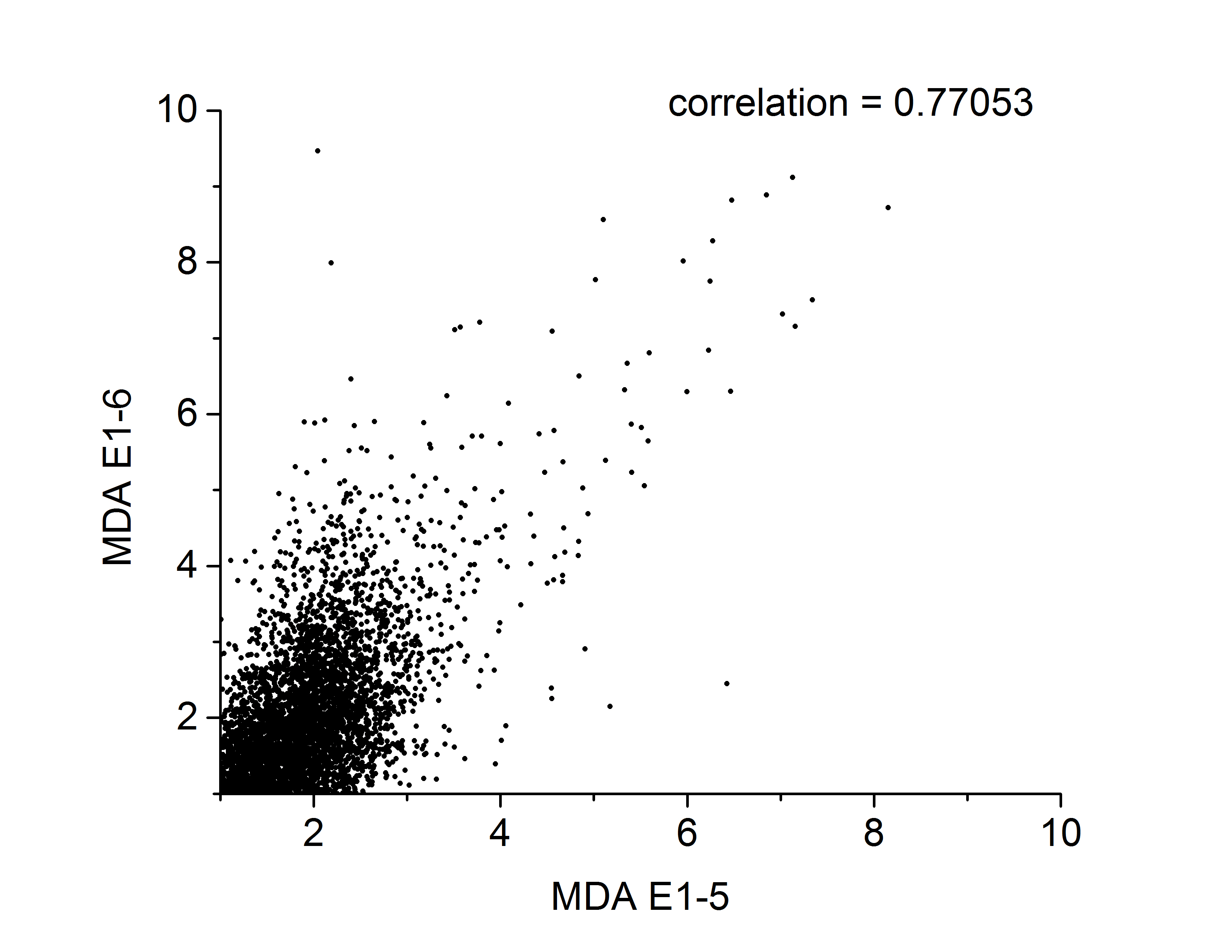 | 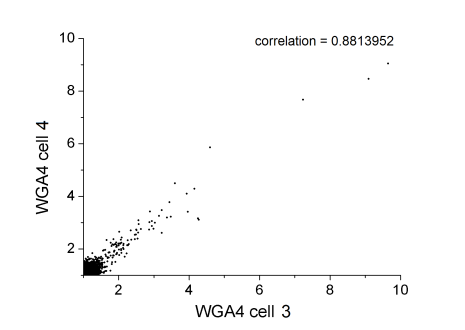 |
| 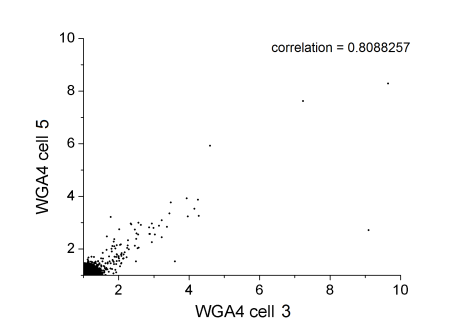 | 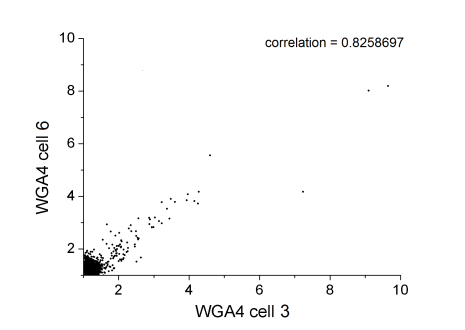 | 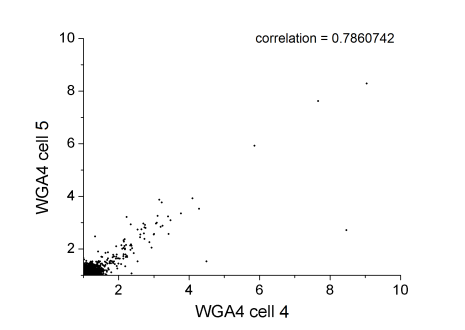 |
| 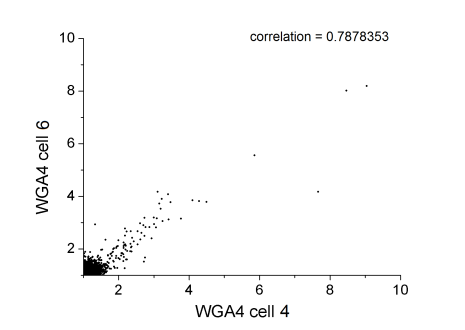 | 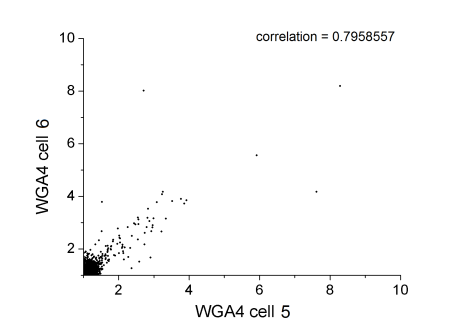 | 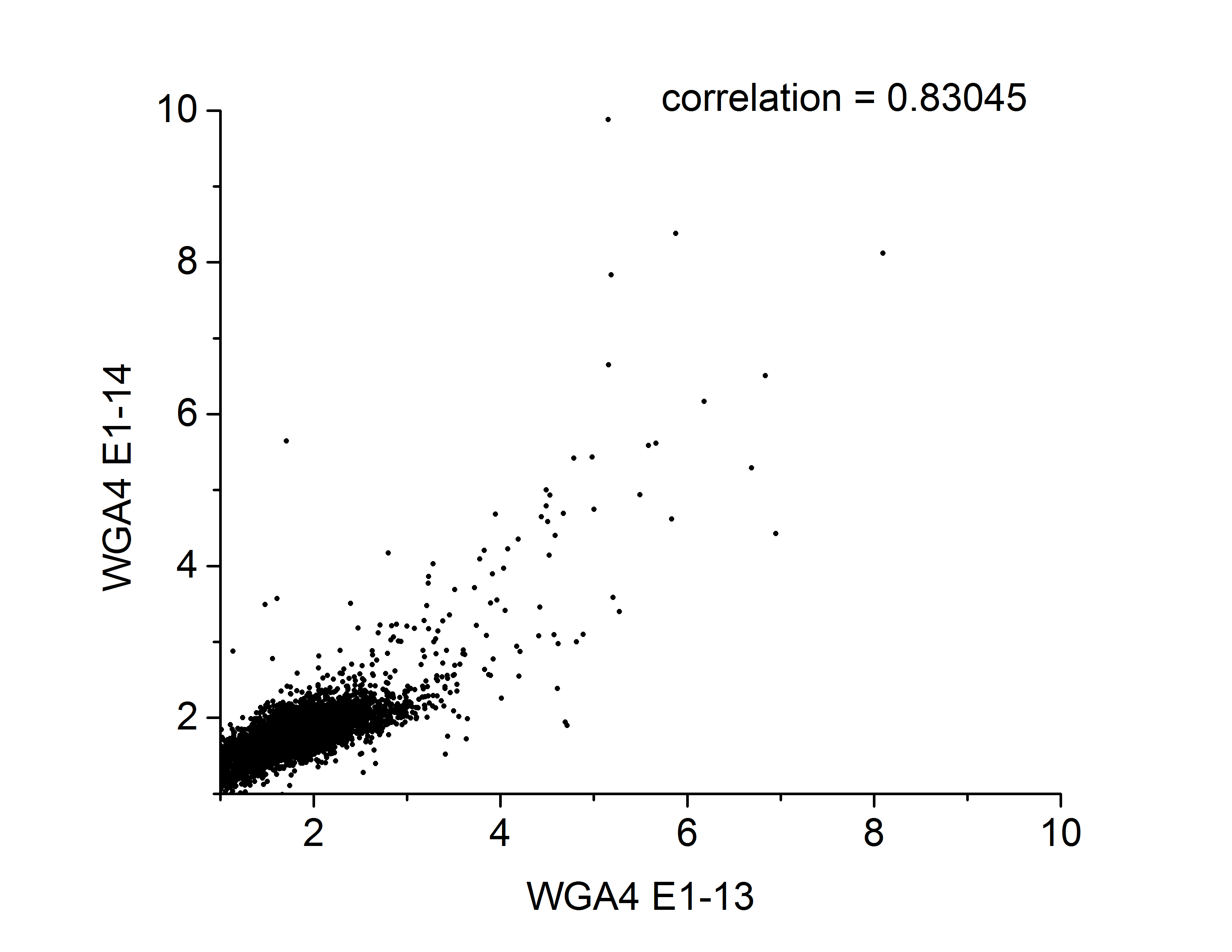 |

**Fig. S2** | Scatter plots showing the reproducibility of three whole genome amplification methods. Details have been described in the method section and all of the cells used in this study have been analyzed here.


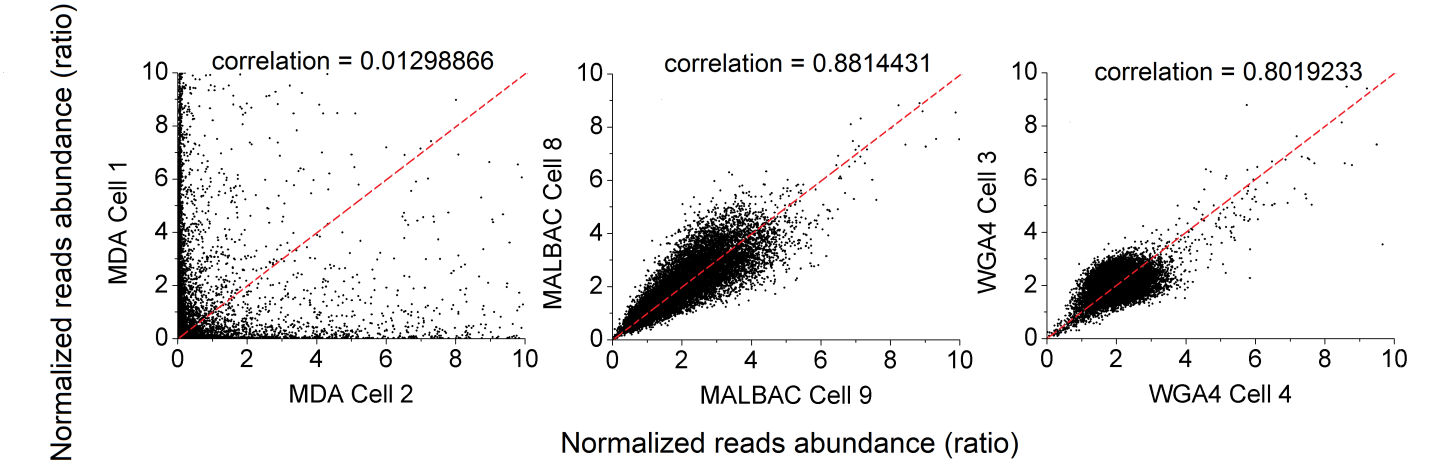


**Fig. S3** | Scatter plots showing the reproducibility of three whole genome amplification methods at 200 kb bin size. Together with **Fig. 4a** in main text, we show that MALBAC and WGA4 have great reproducibility at 500 kb and 200 kb bin size.

| 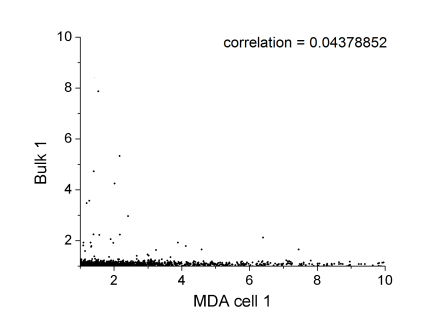 | 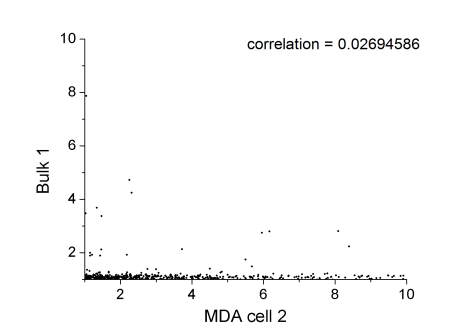 | 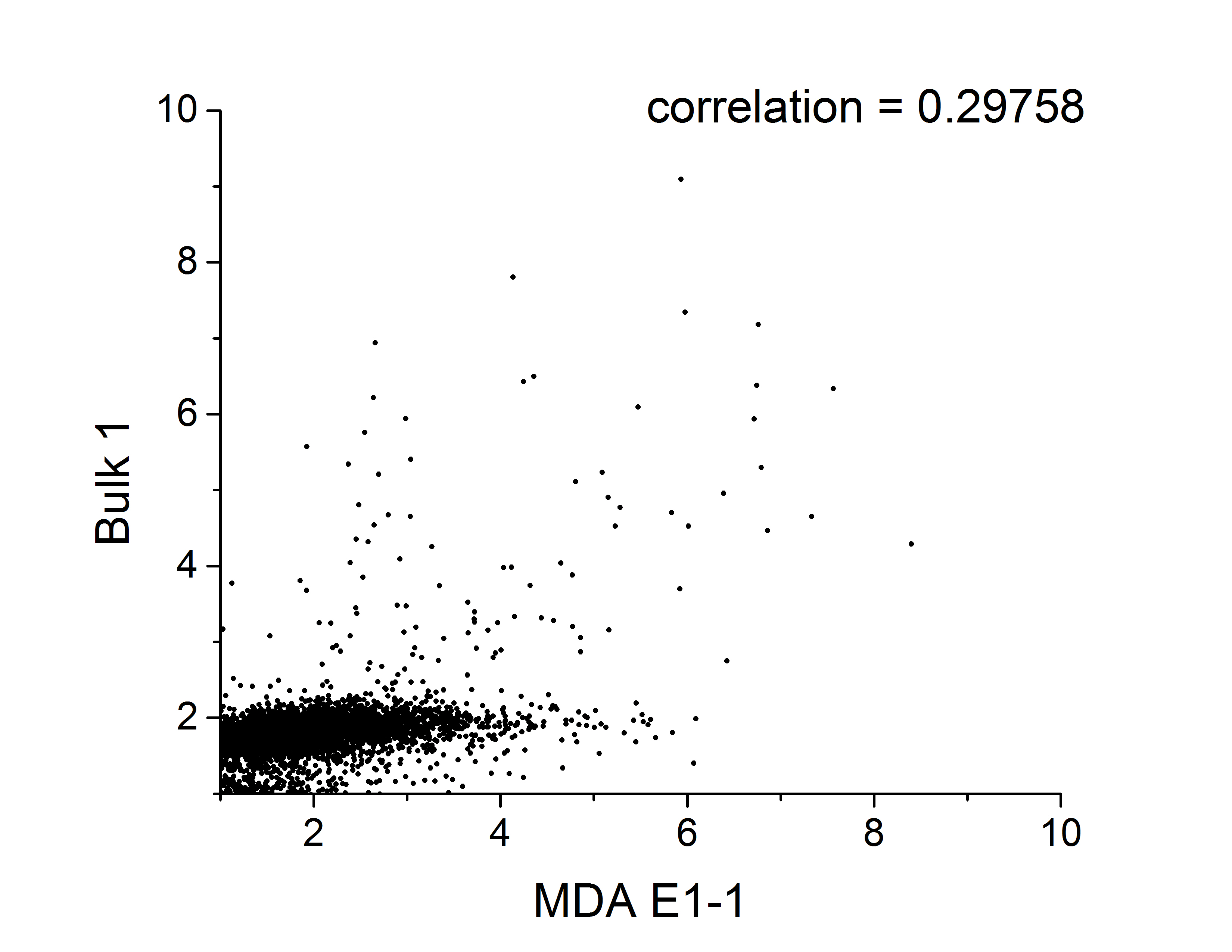 |
| --- | --- | --- |
| 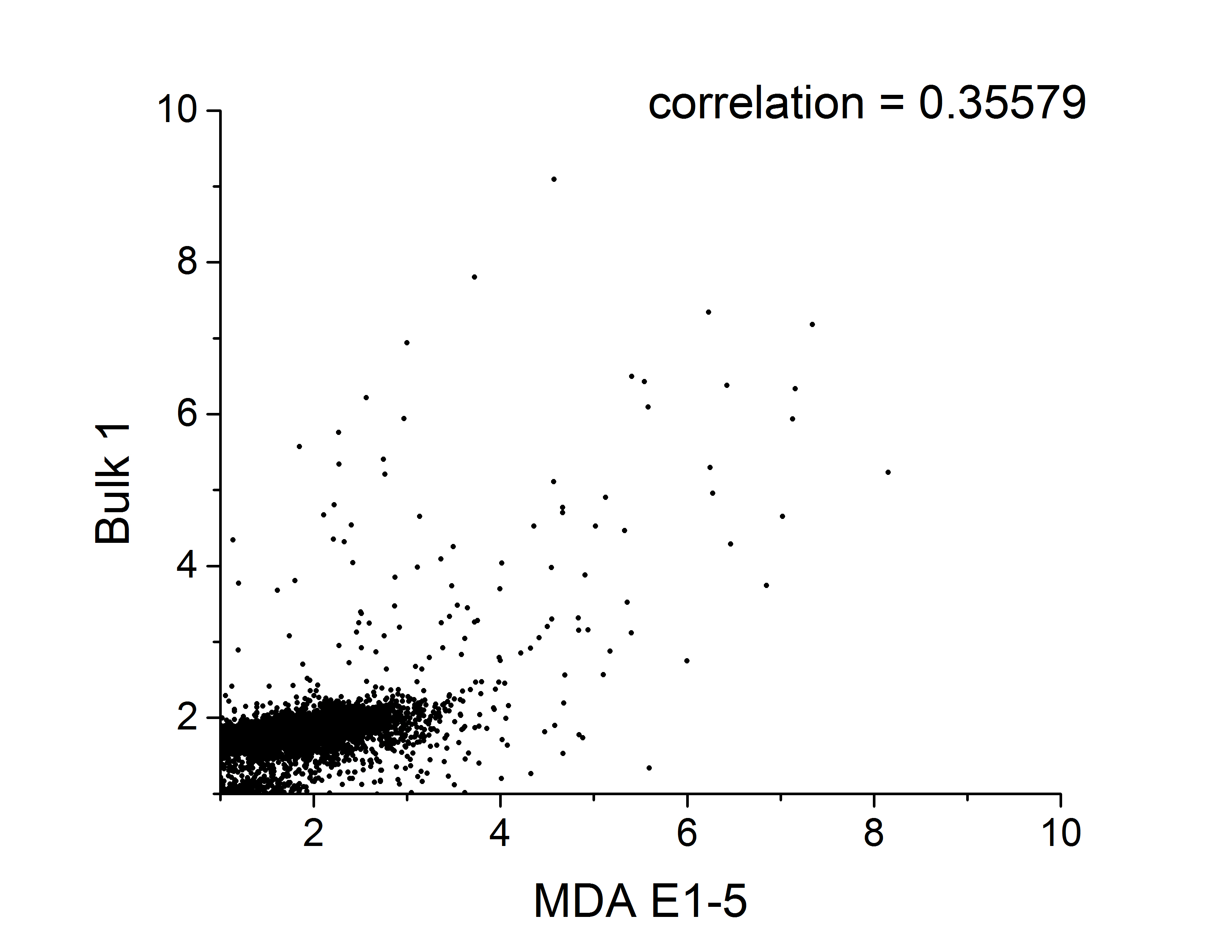 | 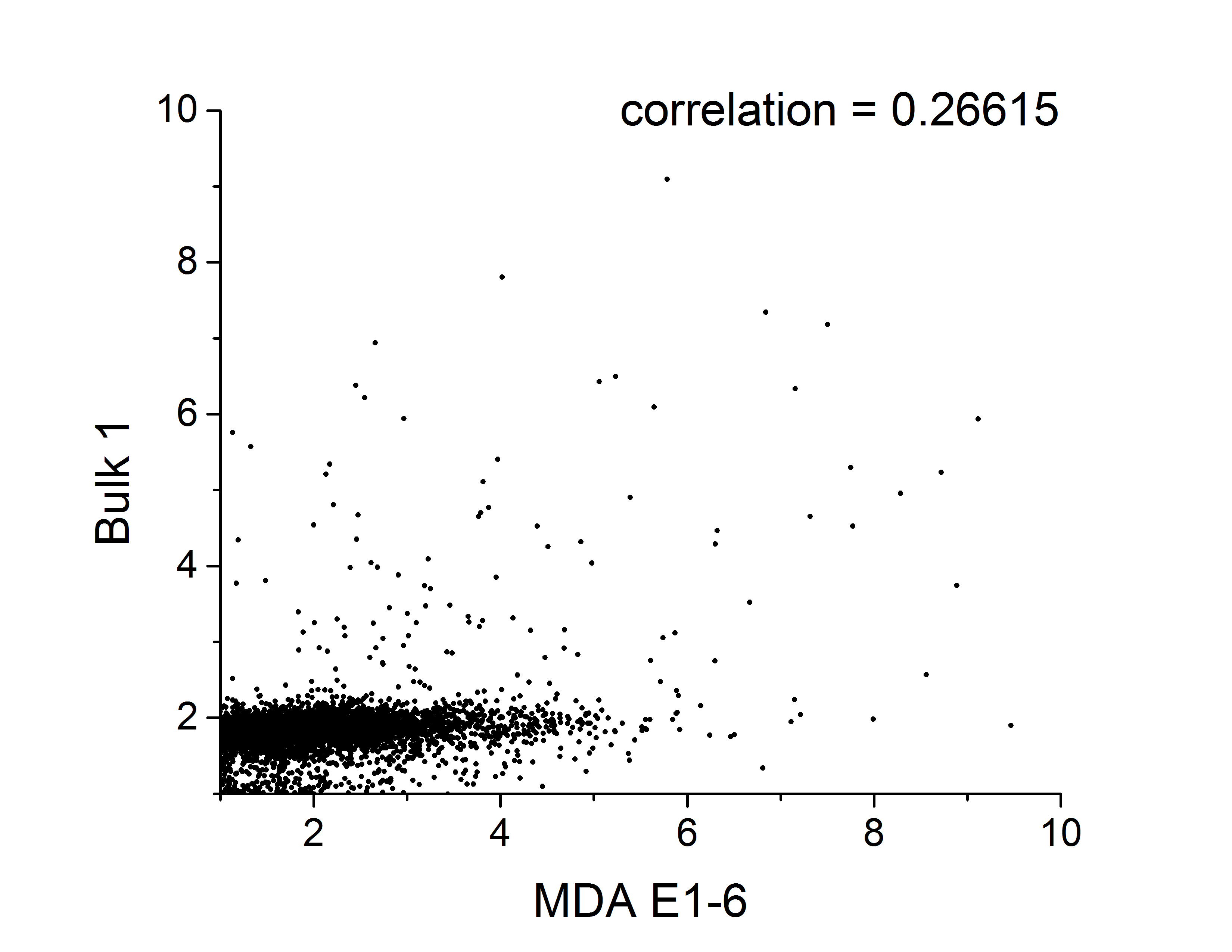 | 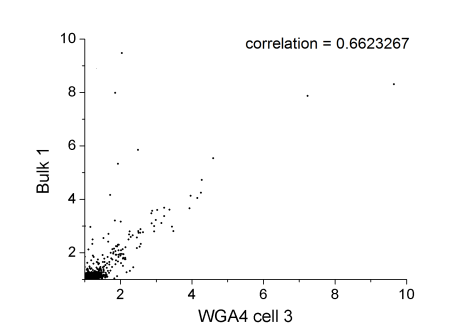 |
| 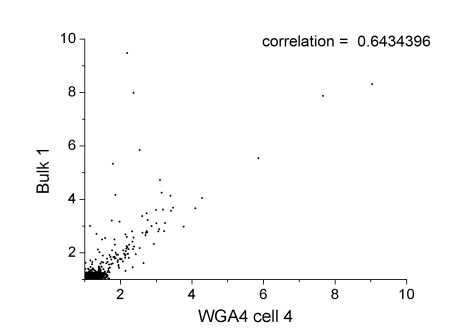 | 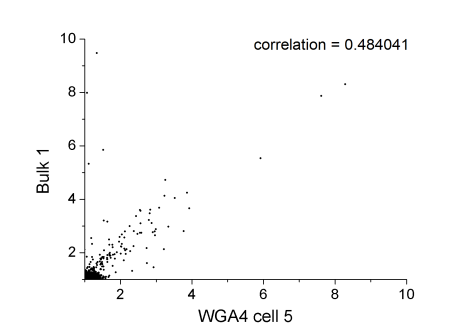 | 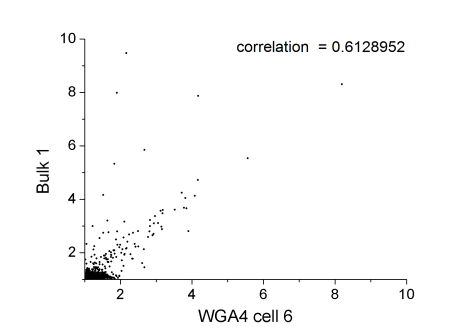 |
| 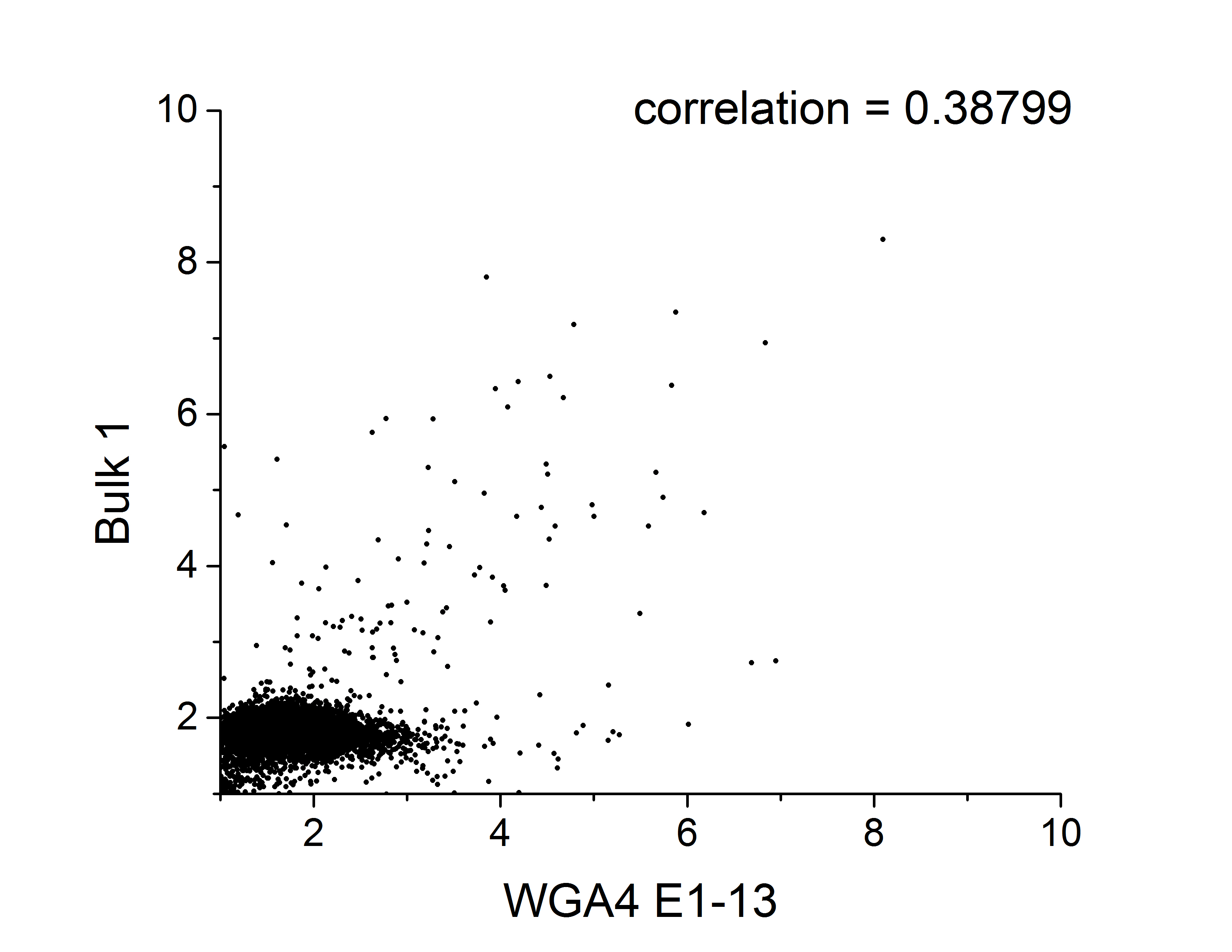 | 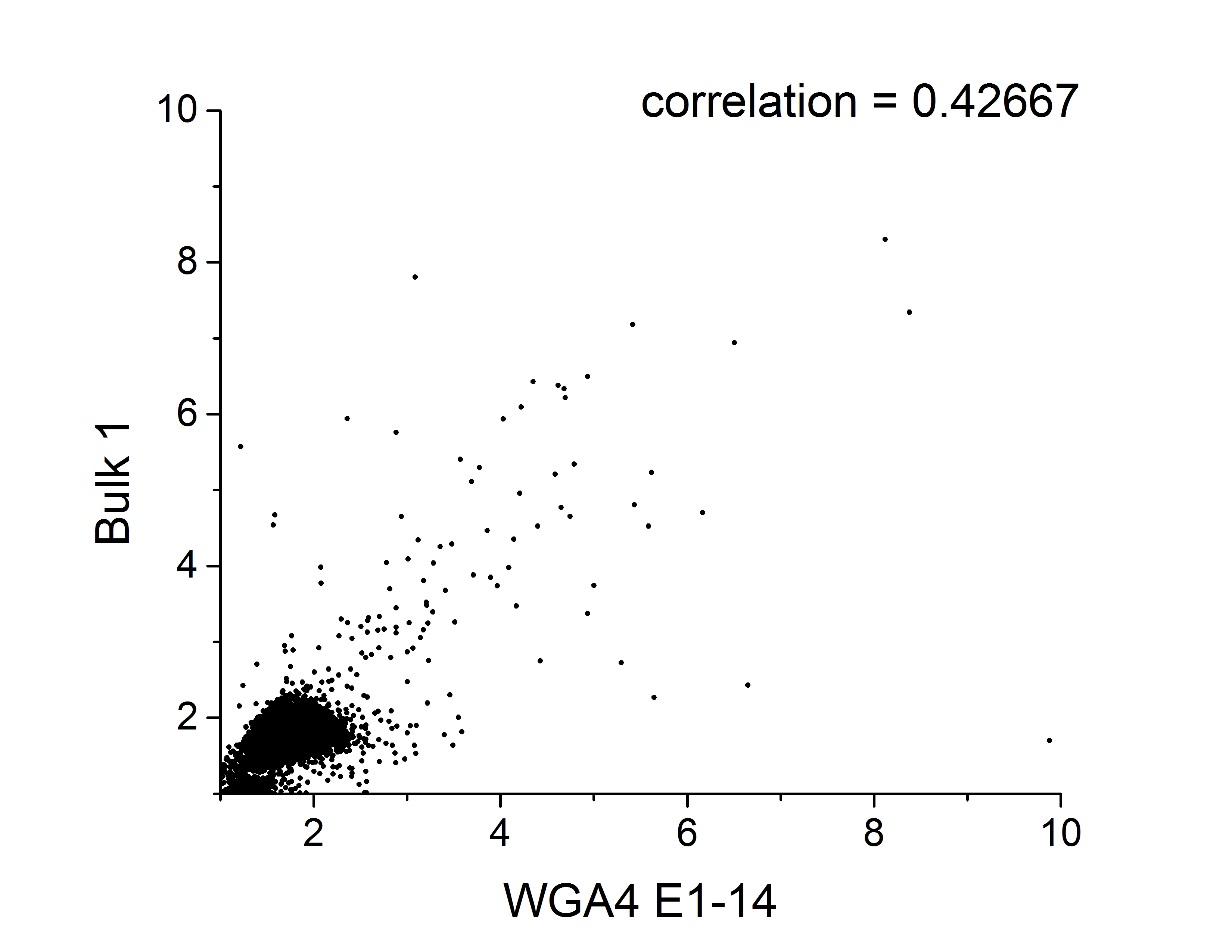 | 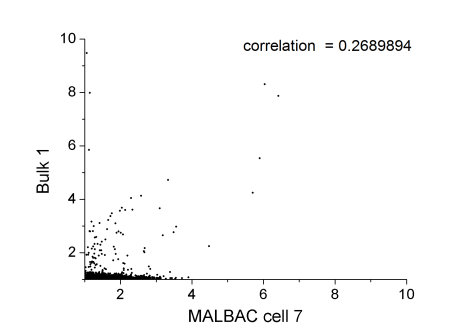 |
| 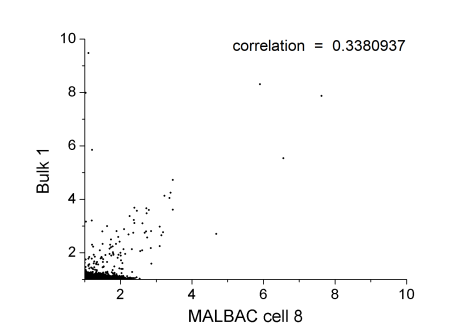 | 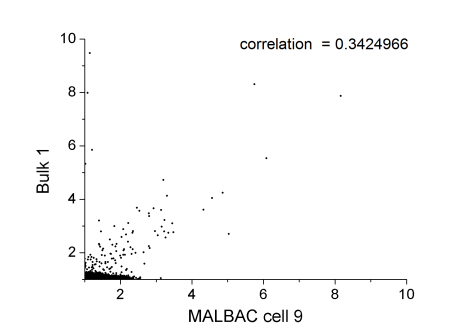 | 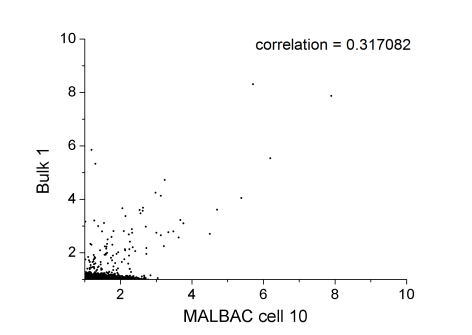 |
| 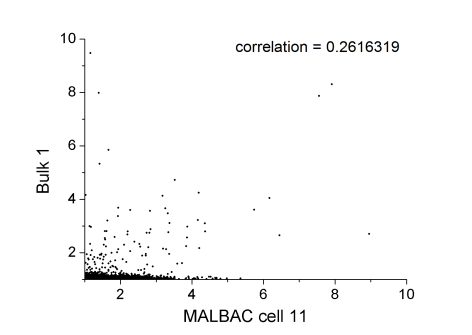 | 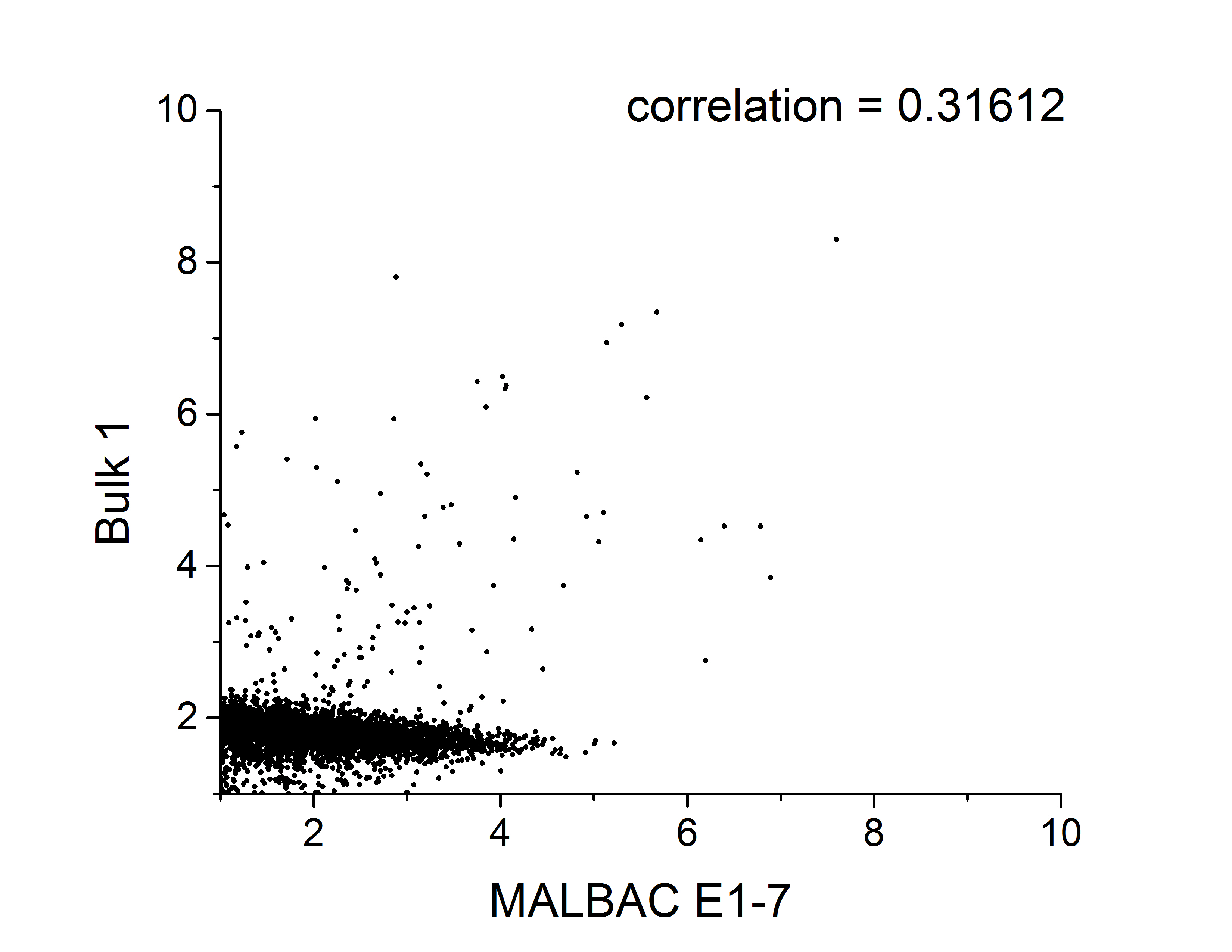 | 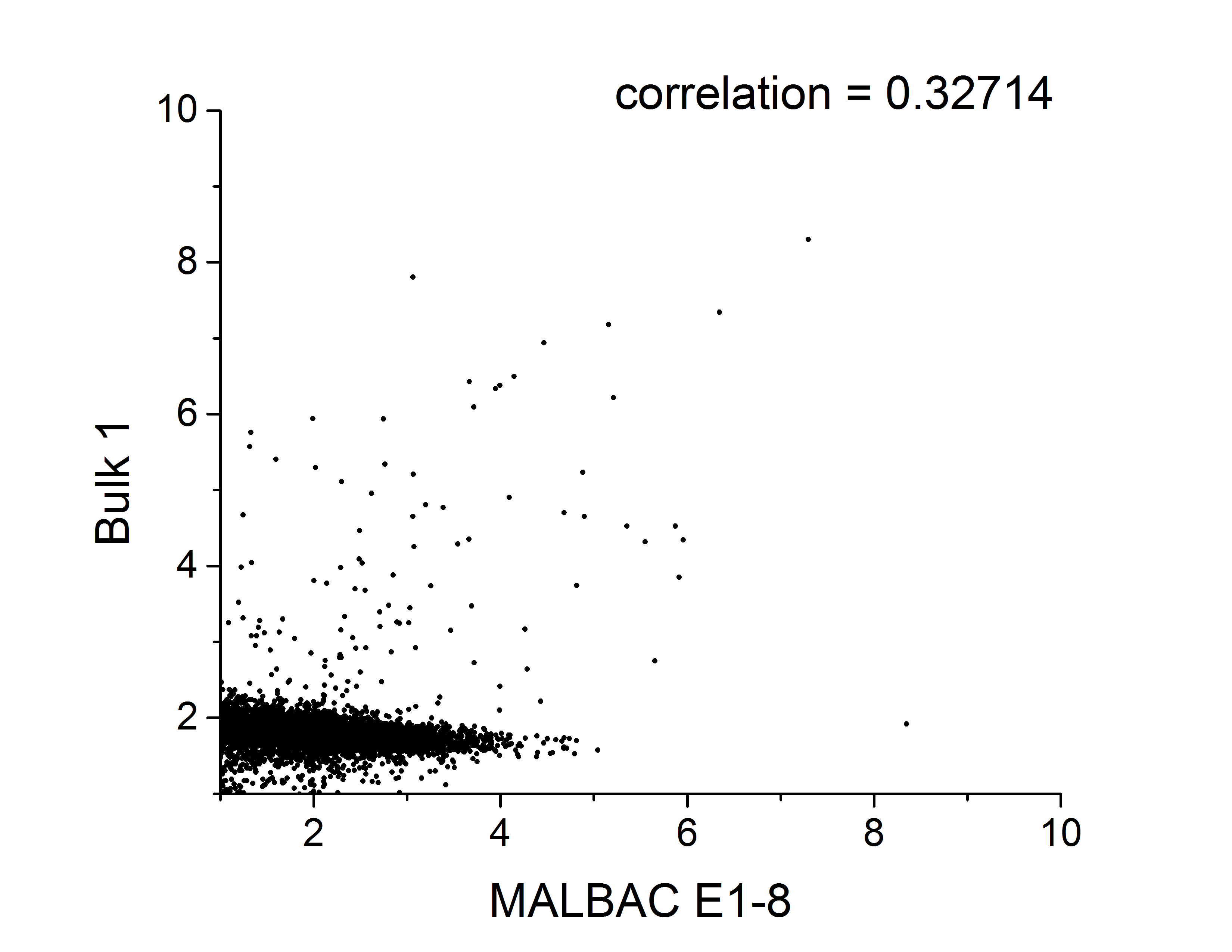 |
| 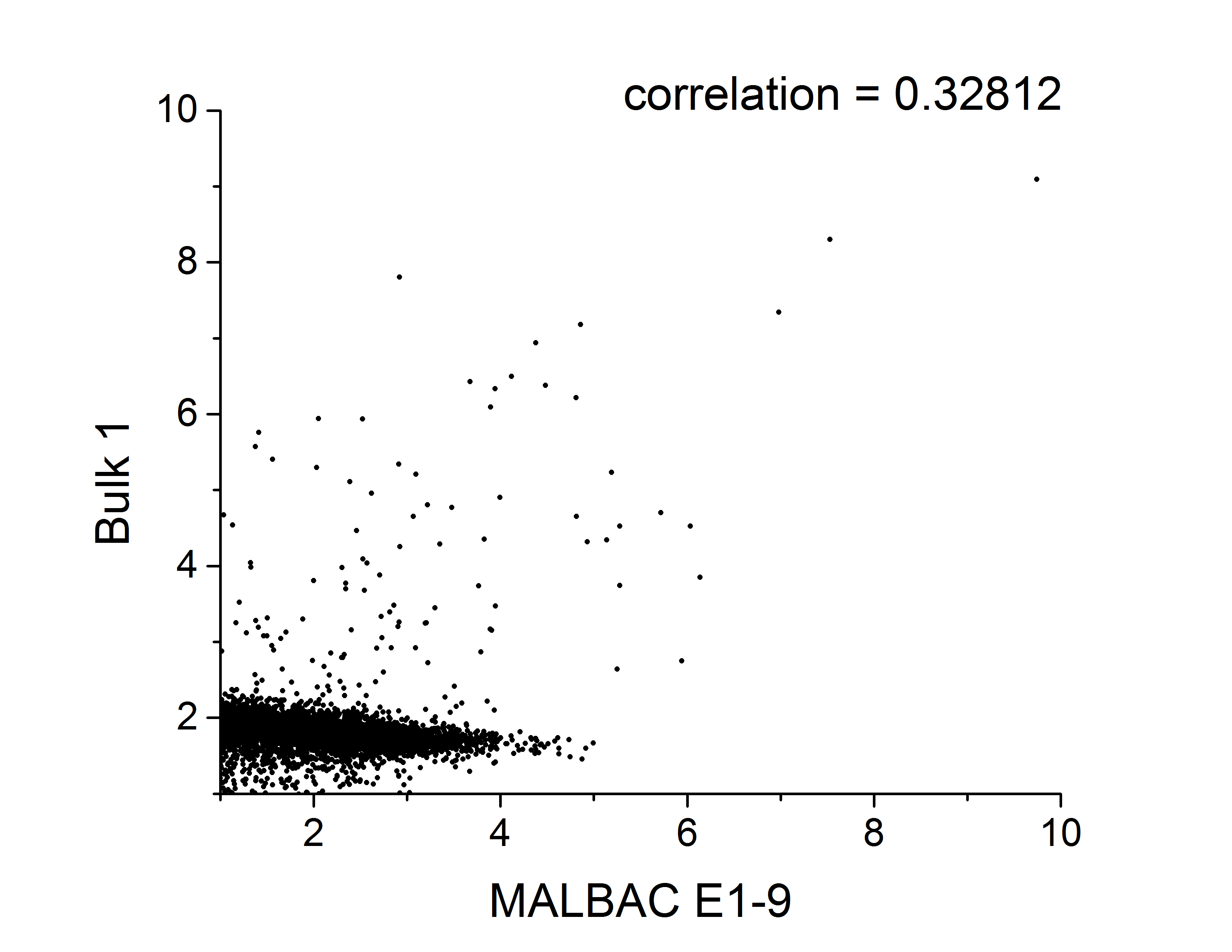 |  |  |

**Fig. S4** | Correlation between three single-cell methods and bulk cell without GC-correction. Details have been described in the method section.

| A | 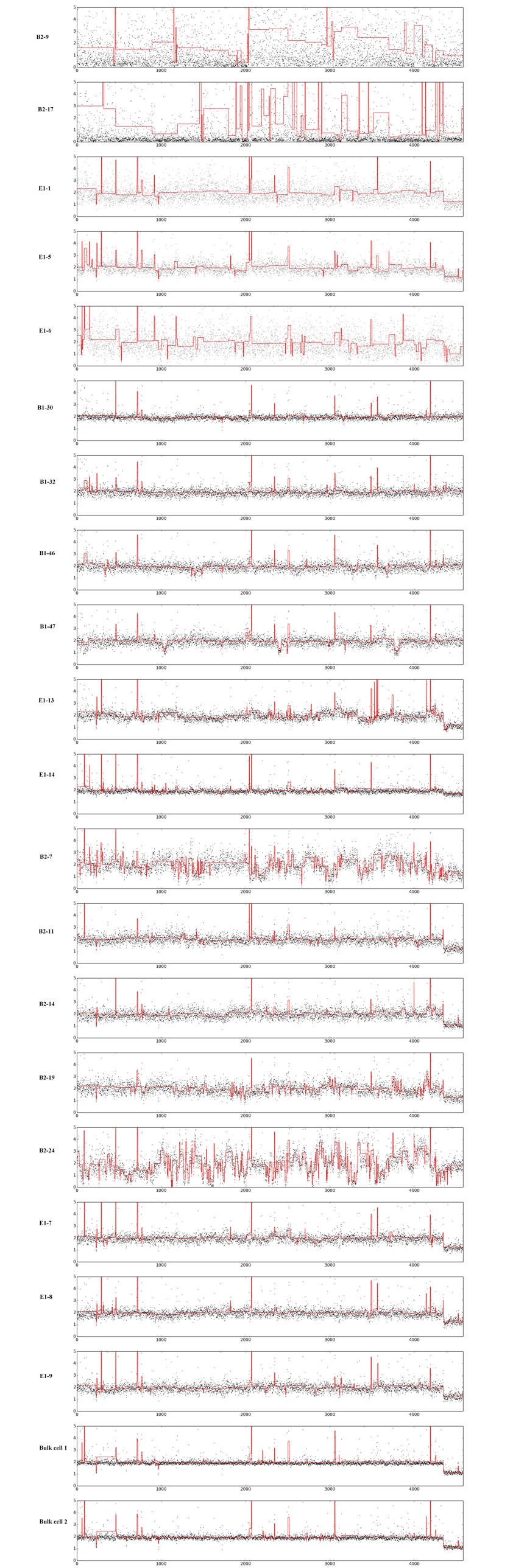 |
| --- | --- |
| B | 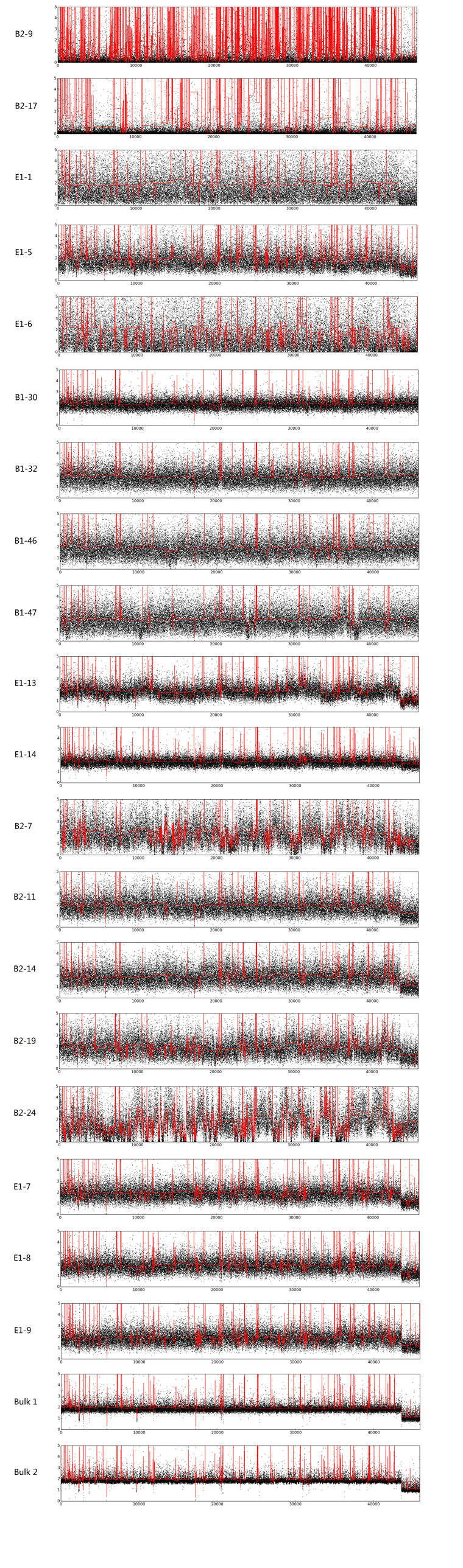 |

**Fig. S5** | Predicted copy numbers of genomic regions after GC-correction (red line) for the first batch and second batch using 500kb bin size (A) and 200kb bin size (B). The copy number of X chromosome is not able to be detected by MDA in the first batch. However, in the second batch, with improved quality of data, the copy number of x chromosome is able to be detected by MDA. We investigated the reason why the second batch has better results than the first batch. The first batch of single cells was isolated and stored in -80 degree for 14 weeks before the MDA amplification performed, while the second batch single cells was isolated, shipped to BGI at Shenzhen and performed MDA amplification immediately. Also, different technicians who performed the MDA amplification may have different results, because the single cell experiment must be handled carefully to avoid contamination.


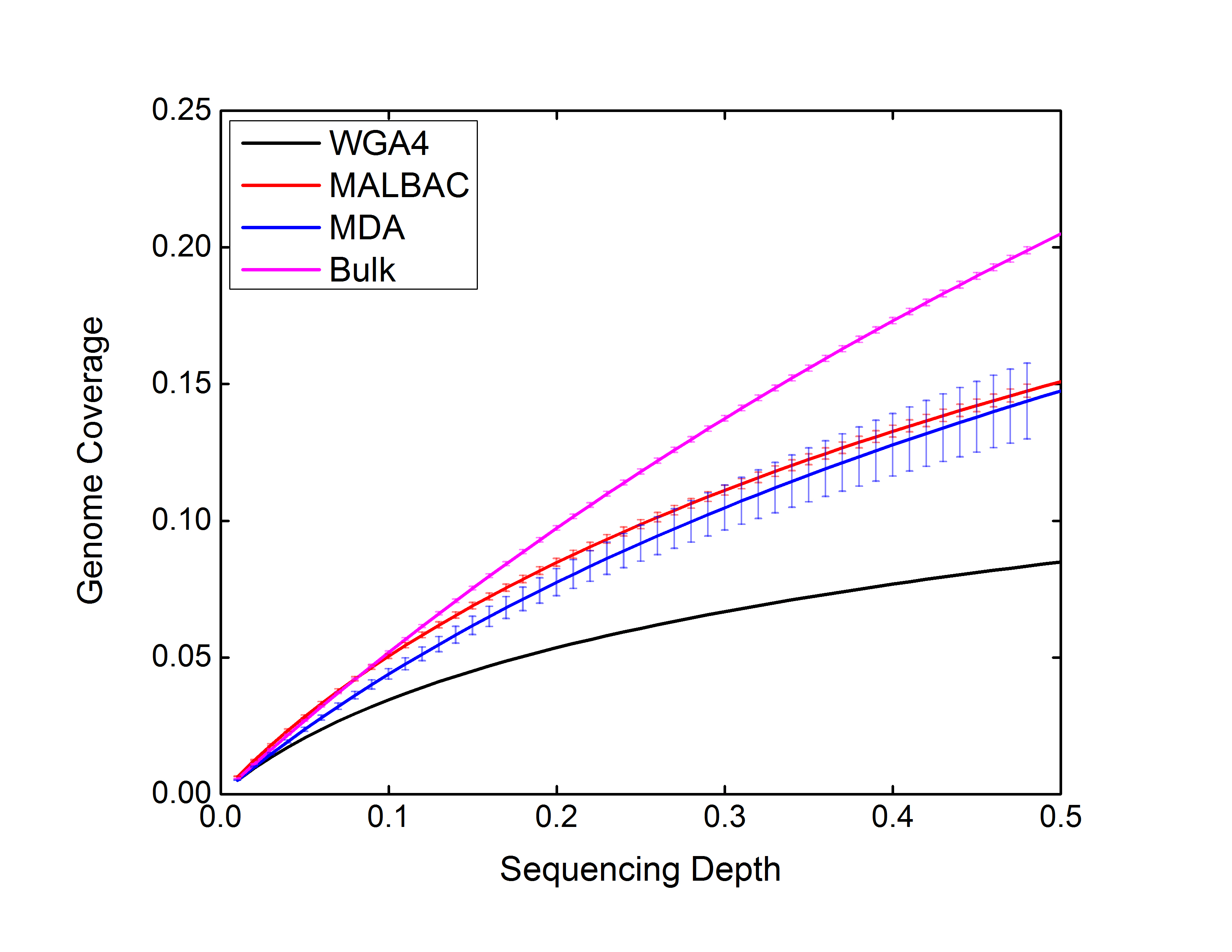


**Fig. S6 |** The relationship between genome coverage and the sequencing depth for all three methods. For each sample, we randomly selected 10% of the reads from the total sequence reads. These reads were aligned to the reference genome. We calculated the percentage of the genome that was covered by at least one read. We repeated the sampling 10 times and determined the average genome coverage at 10% sampling depth. We did this by selecting 20%, 30%, . . . , and 90% reads. At different sampling depths, the same process was followed to generate the information shown. We performed sampling to each sample and calculated the genome coverage for each sample. The error bar is standard error over samples with the same method. For MDA method, only cells from the second batch are included. For WGA4, only cell 5 has reached the sequencing depth 0.5X, therefore, only cell 5 is included in this figure.
